# Dimensional accuracy of 3D printing navigation templates of chemical-based sterilisation

**DOI:** 10.1038/s41598-022-05412-7

**Published:** 2022-01-24

**Authors:** Wenxi Zhang, Xia Lin, Junfeng Jiang

**Affiliations:** 1 Department of Orthopedics, The Peoples’ Hospital of Liyang, Changzhou, China; 2 Department of Disinfection Supply, The Peoples’ Hospital of Liyang, Changzhou, China; 3 College of IOT Engineering, Hohai University, Changzhou, China

**Keywords:** Characterization and analytical techniques, Preclinical research, Translational research

## Abstract

3D printed navigational templates have facilitated the accurate treatment of orthopaedic patients. However, during practical operation, it is found that the location hole occasionally deviates from the ideal channel. As such, there will be a security risk in clinical applications. The purpose of this study was to evaluate the influence of chemical-based sterilisation methods on the dimensional accuracy of different materials and the influence of module parameters on the degree of deformation. We found that polylactic (PLA) modules sterilised with ethylene oxide (EO) would undergo micro-deformation, and these micro-deformation characteristics depend on the building direction, i.e., the module stretches in the Z direction and shrinks in the X and Y directions. Heat-resisting polylactide (HR-PLA) has the same melting temperature (*T*_*m*_) as PLA, but its glass transition temperature (*T*_*g*_) is greater than the EO sterilisation temperature, so there is no obvious deformation after EO sterilisation. The layer height of the module were inversely proportional to the degree of deformation in the same sterilisation method. The deformation time of the module is concentrated within 2 h after heating. The micro-deformation of the 3D printing module depends on its *T*_*g*_, sterilisation temperature, and duration of the sterilisation cycle.

## Introduction

In recent years, digital orthopaedics technology applied in the field of surgery has developed rapidly; the maturing of 3D printing technology has meant that it has become increasingly widely used in orthopedics. A 3D printed navigational template allows an operation to be more accurate, such as the use of a pedicle screw navigational template (PSNT) in a spinal operation. Particularly in the placement of an upper cervical spine pedicle screw, accuracy can be significantly increased^1–3^. However, the design of the navigation template requires a certain anatomical basis for the ideal design of the positioning hole, and the required test data are generally based on a model or a corpse^4–7^. When applied in a real operation, the use of the template is often affected due to the range of the stripping. Despite the optimisation of the template design, deviations still arise.

Although the machining precision of a 3D printed navigational template is higher than the accuracy required in a clinic, accuracy problems sometimes still appear during an operation. Some techniques have been developed to solve this problem. Experts in different fields look for deviation from each detail by using computer tomography (CT) to scan anatomical specimens, by using stereolithography appearance (SLA) to print multiple models for fixed coordinate measurement^8^, and by using various printing methods^9^ to compare the dimensional accuracy of models. Studies have found that the absolute value of the deviation is so small that it does not affect clinical applications. To find the reasons for this deviation, researchers have carried out tests from other details using techniques such as image acquisition, image segmentation, and stereolithography (STL) transformation^10–13^. It has been found that an STL file obtained by people with different experiences shows large differences that greatly influence the final accuracy of the 3D model.

This requirement to sterilise a 3D printed navigation template further raises concerns about the dimensional accuracy of a navigation template. If the navigation template is deformed or modified during the sterilisation procedure, it will affect the accuracy of a surgical procedure. Whether the sterilisation affects the 3D printed navigation template or not is a topic which has not been addressed. Sterilisation is a process that eliminates all forms of microbial life, such as fungi, bacteria, and viruses resulting from processing and handling^14^. However, sterilisation can affect the material's surface and lead to changes in physical–chemical, morphological, mechanical, chemical, and biological features^15^. Chemical-based, including plasma and ethylene oxide (EO), and radiation-based sterilisation methods (gamma and electron-beam) are the most commonly used sterilisation tools for a wide variety of polymers and polymeric scaffolds. Changes in *T*_*g*_ predict how chemical and structural changes due to sterilisation can affect the chain mobility and thus the polymer properties. The *T*_*g*_ of polylactic (PLA) decreased after any of the sterilisation methods were employed; the chemical sterilisation method had an even stronger effect and reduced *T*_*g*_ even more. Interestingly, in polymers containing higher polyethylene glycol (PEG) and desaminotyrosyl-tyrosine, all sterilisation methods increased *T*_*g*_^16^. Polymers that contain a high proportion of PEG and carboxylic acid groups can only be sterilised when using mild conditions. Likewise, the tendency of highly degradable polymers (those with high PEG and carboxylic acid content) to undergo hydrolytic cleavage during EO and plasma exposure, limits the use of these chemical sterilisation methods^17^.

The overall goal of this work was to evaluate the effect of chemical-based sterilisation methods on the dimensional accuracy of different materials and to get at the effect of module parameters on the degree of deformation. In recent years, research on the reliability of the navigation template after sterilisation has gradually increased. The divergence of the research is whether the navigation template is reliable after high-pressure steam sterilisation^18–22^, but safety of chemical-based sterilisation is unanimously recognised. Different from previous studies, we found that chemical-based sterilisation also had a risk of deformation, and the deformation was related to the parameters of the module. The key contribution of this work is to provide a solution for reliable clinical applications of navigation templates.

## Materials and methods

### Materials and test bodies design

**Figure 1:**
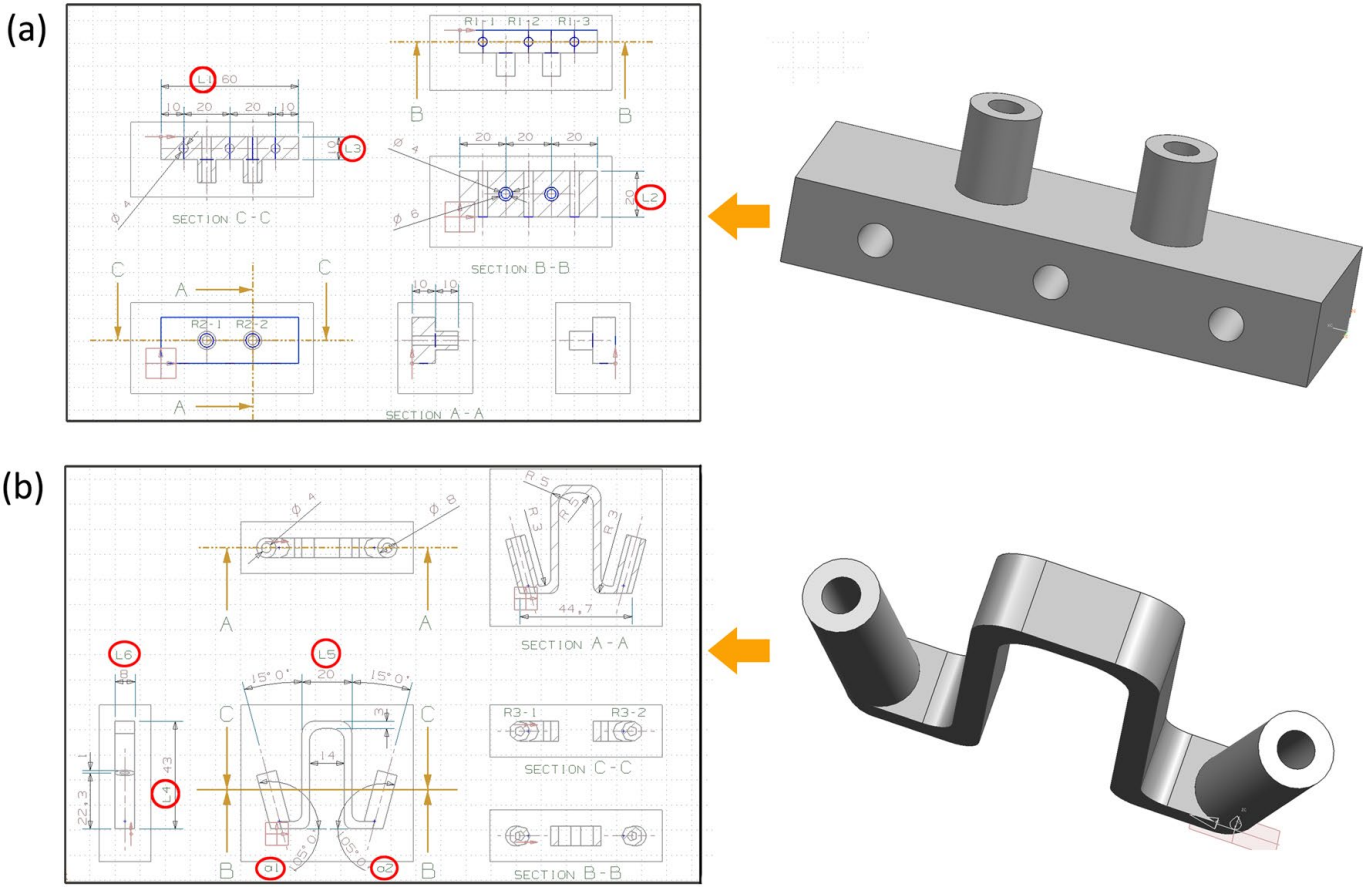
Module and PSNT design: (**a**) symbol definition of the view and section of the test module; (**b**) symbol definition of view and section of PSNT. The circles indicate where parameters are measured.

Unigraphics NX 4.0 software (Siemens PLM Software, Germany) and Cura software (Ver 5.02.1, Ultimaker, Netherlands) was used, and a test module was designed with five Φ 4 mm cross-location holes (Fig. 1a), with a length of 60 mm, a width of 20 mm, and a thickness of 10 mm. A PSNT was designed with a location pole at an angle of 15° and a pore diameter of 4 mm (Fig. 1b). The additive manufacturing (AM) methods used were fused deposition modelling (FDM) printing (iMaker4xxl, Hisen Tech, China), selective laser sintering (SLS) printing (SLS402, ZRapid Tech, China), and SLA printing (SLA600, ZRapid Tech, China). The printing materials used were acrylonitrile butadiene styrene (ABS), PLA, high-toughness polylactide (HT-PLA), heat-resisting polylactide (HR-PLA), photosensitive resin, and polyamide (PA). The subtractive manufacturing (SM) methods used were computer numerical control (CNC) and injection moulding, and the materials used were all ABS for this type of manufacturing method. Methods of sterilisation used were high-pressure steam (GEB 6617, Getinge Group, Sweden), EO (3 M Steri-Vac™ Steriliser/Aerator Model 8XL, USA), and plasma sterilisation (STERRAD100S, Johnson & Johnson Inc, USA). The time–temperature characteristic of the material was measured using a constant temperature drying oven (101-0B, Supo Instrument Inc, China).

This study is a crucial part of clinical trial. All experimental protocols were approved by the People’s Hospital of Liyang, all methods were carried out in accordance with relevant guidelines and regulations, and written informed consent was obtained from all subjects.

### Assessment of dimensional accuracy based on two low-temperature sterilisation methods and two building directions

The modules designed by us (Fig. 1a) were printed using FDM, and two building directions were used. The printing infill densities were 20%, 50%, and 100%, with a layer height of 0.1 mm, a nozzle of Φ 0.4 mm, PLA material, and a nozzle temperature of 204 °C. 3D printing was used to generate a total of 12 modules, the serial numbers of which were n – 1, n – 2, n – 3, and n-4 (n = 1, 2, and 3). See Table 1 for detailed parameters. This experiment involved two questions: (1) Would low-temperature sterilisation result in a significant change in dimensions of the 3D printed module? (2) Would the changing characteristic of the module be related to the building direction?

**Table 1:** Parameters and sterilisation methods of all test modules.

Serial number	Material	Layer height (mm)	Infill (%)	Bed temp (°C)	Extrusion temp (°C)	Cylindrical direction	Filament diameter (mm)	Sterilisation method	Amount
M1	HT-PLA	0.1	50	50	208	Parallel to Z-axis	1.75	EO	10
M2	HT-PLA	0.2	50	50	208	Parallel to Z-axis	1.75	EO	10
M3	HT-PLA	0.3	50	50	208	Parallel to Z-axis	1.75	EO	10
M4	HT-PLA	0.2	10	50	208	Parallel to Z-axis	1.75	EO	10
M5	HT-PLA	0.2	20	50	208	Parallel to Z-axis	1.75	EO	10
M6	HT-PLA	0.2	30	50	208	Parallel to Z-axis	1.75	EO	10
M7	HT-PLA	0.3	50	50	208	Parallel to Z-axis	1.75	EO	10
M8	HT-PLA	0.3	50	50	208	Parallel to Z-axis	1.75	EO	10
M9	HT-PLA	0.3	50	50	208	Parallel to Z-axis	1.75	EO	10
M10	HT-PLA	0.3	50	50	208	Parallel to Z-axis	1.75	EO	10
1–1	PLA	0.1	20	50	204	Parallel to Z-axis	1.75	EO	1
1–2	PLA	0.1	20	50	204	Parallel to Z-axis	1.75	Plasma	1
1–3	PLA	0.1	20	50	204	Parallel to Y-axis	1.75	EO	1
1–4	PLA	0.1	20	50	204	Parallel to Y-axis	1.75	Plasma	1
2–1	PLA	0.1	50	50	204	Parallel to Z-axis	1.75	EO	1
2–2	PLA	0.1	50	50	204	Parallel to Z-axis	1.75	Plasma	1
2–3	PLA	0.1	50	50	204	Parallel to Y-axis	1.75	EO	1
2–4	PLA	0.1	50	50	204	Parallel to Y-axis	1.75	Plasma	1
3–1	PLA	0.1	100	50	204	Parallel to Z-axis	1.75	EO	1
3–2	PLA	0.1	100	50	204	Parallel to Z-axis	1.75	Plasma	1
3–3	PLA	0.1	100	50	204	Parallel to Y-axis	1.75	EO	1
3–4	PLA	0.1	100	50	204	Parallel to Y-axis	1.75	Plasma	1
6–1	ABS	0.2	50	100	260	In the X–Z plane	1.75	EO	1
6–2	ABS	0.2	50	100	260	In the X–Z plane	1.75	Autoclaving	1
6–3	ABS	0.2	50	100	260	In the X–Z plane	1.75	Plasma	1
7–1	Nylon	0.1	100	190	N/A	In the X–Y plane	N/A	EO	1
7–2	Nylon	0.1	100	190	N/A	In the X–Y plane	N/A	Autoclaving	1
7–3	Nylon	0.1	100	190	N/A	In the X–Y plane	N/A	Plasma	1
8–1	Resin	0.05	100	N/A	N/A	In the X–Y plane	N/A	EO	1
8–2	Resin	0.05	100	N/A	N/A	In the X–Y plane	N/A	Autoclaving	1
8–3	Resin	0.05	100	N/A	N/A	In the X–Y plane	N/A	Plasma	1
0–1	ABS	N/A	100	N/A	N/A	N/A	N/A	EO	1
0–2	ABS	N/A	100	N/A	N/A	N/A	N/A	Autoclaving	1
9–1	ABS	N/A	100	N/A	N/A	N/A	N/A	EO	1
9–2	ABS	N/A	100	N/A	N/A	N/A	N/A	Autoclaving	1
A1–A10	PLA	0.2	20	50	204	Parallel to Z-axis	1.75	EO	10
A11–A20	PLA	0.2	20	50	204	Parallel to Y-axis	1.75	EO	10
B1–B10	PLA	0.2	20	50	204	Parallel to Z-axis	2.85	EO	10
B11–B20	HR-PLA	0.2	20	50	204	Parallel to Z-axis	1.75	EO	10
C1–C12	PLA	0.1	50	50	204	In the X–Y plane	1.75	EO	12

### Measurement of deformation characteristics based on different printing parameters

The printing infill densities of modules were 10%, 20%, 30%, and 50%, with a layer height of 0.1 mm, 0.2 mm and 0.3 mm, a nozzle of Φ 0.4 mm, HT-PLA material, and a nozzle temperature of 208 °C. 3D printing was used to generate a total of 60 modules, the serial numbers of which were M1, M2, M3, M4, M5, and M6. We marked the midpoint of each side of each module as the test point. There were 10 sets of data for each serial number with the values being the means ± SD. All modules were EO sterilised (Table 1). This experiment involved a question: How would printing parameters affect the module dimension after EO sterilisation?

### Time–temperature curve of M series modules

The printing infill densities of modules were 50%, with a layer height of 0.3 mm, a nozzle of Φ 0.4 mm, HT-PLA material, and a nozzle temperature of 208 °C. A total of 40 modules were printed, the serial numbers of which were M7, M8, M9, and M10. Similarly, we marked the midpoint of each side of each module as the test point. There were 10 sets of data for each serial number. A constant temperature drying oven was used. The module M7 was measured at a constant temperature of 55 °C, and the measurement time nodes were 0 h, 2 h, 4 h, and 6 h. Similarly, M8, M9, and M10 were measured at 65 °C, 75 °C, and 45 °C. This experiment involved two questions: (1) How strong is the influence of different temperatures in dimensional accuracy of the module? (2) Is it related to time?

### Combination test of PSNT using three materials and three sterilisation methods

In this experiment, we designed a PSNT (Fig. 1b); ABS material was printed using the FDM method, photosensitive resin material was printed using the SLA method, and nylon material was printed using the SLS method. The serial numbers were 6 – n, 7 – n, and 8 – n (n = 1, 2, and 3). After recording data for each print model, three types of sterilisation were used: EO, plasma, and high-pressure steam sterilisation (Table 1). The dimensions of each PSNT before and after sterilisation were compared. This experiment involved a question: Would the deformation of PSNT using three materials other than PLA, follow the rules?

### Measurement of five groups of modules before and after EO sterilisation

Two sets of 3D printers with different filament diameters (1.75 mm and 2.85 mm) were used to print 10 modules each using a vertical cylindrical method, with serial numbers A1–A10 (group A) and B1–B10 (group B). Ten modules were printed using a horizontal cylindrical method, with serial numbers A11–A20 (group C). Ten modules were printed using HR-PLA of 1.75 mm diameter, with serial numbers B11–B20 (group D). Modules of groups A, B, C, and E were made of PLA material. Twelve PSNTs were printed with serial numbers C1–C12 (group E). Modules A1–A20, B1–B20, and C1–C12 underwent EO sterilisation, see Table 1 for details. This experiment involved a question: would the overall deformation characteristics of the batch of modules still follow the previous law?

### Statistical treatment

IBM SPSS 20.0 software (SPSS Company, USA) was used for statistical data processing. Measurement data were expressed as $\overline{x} \pm s$, and a comparison of the lengths and angles was done using a paired *t*-test in pre- and post-sterilisation measurements within the same group. A one-way ANOVA test was used to compare the difference in dimensional changes between groups (or modules); if the homogeneity of the variance was not satisfied, Welch’s correction to ANOVA was used. It was based on bilateral α = 0.05 inspection level and *p* < 0.05 was considered to be statistically significant.

### Ethical approval

We can confirm that Chinese Clinical Trial Registry Approval was given (ID: ChiCTR1900023259), and that the patients undergoing treatment gave their informed consent to the collection and use of data for research purposes. The privacy rights of human subjects always are observed.

## Results

### Micro-deformation of modules based on two low-temperature sterilisation methods and two building directions

**Figure 2:**
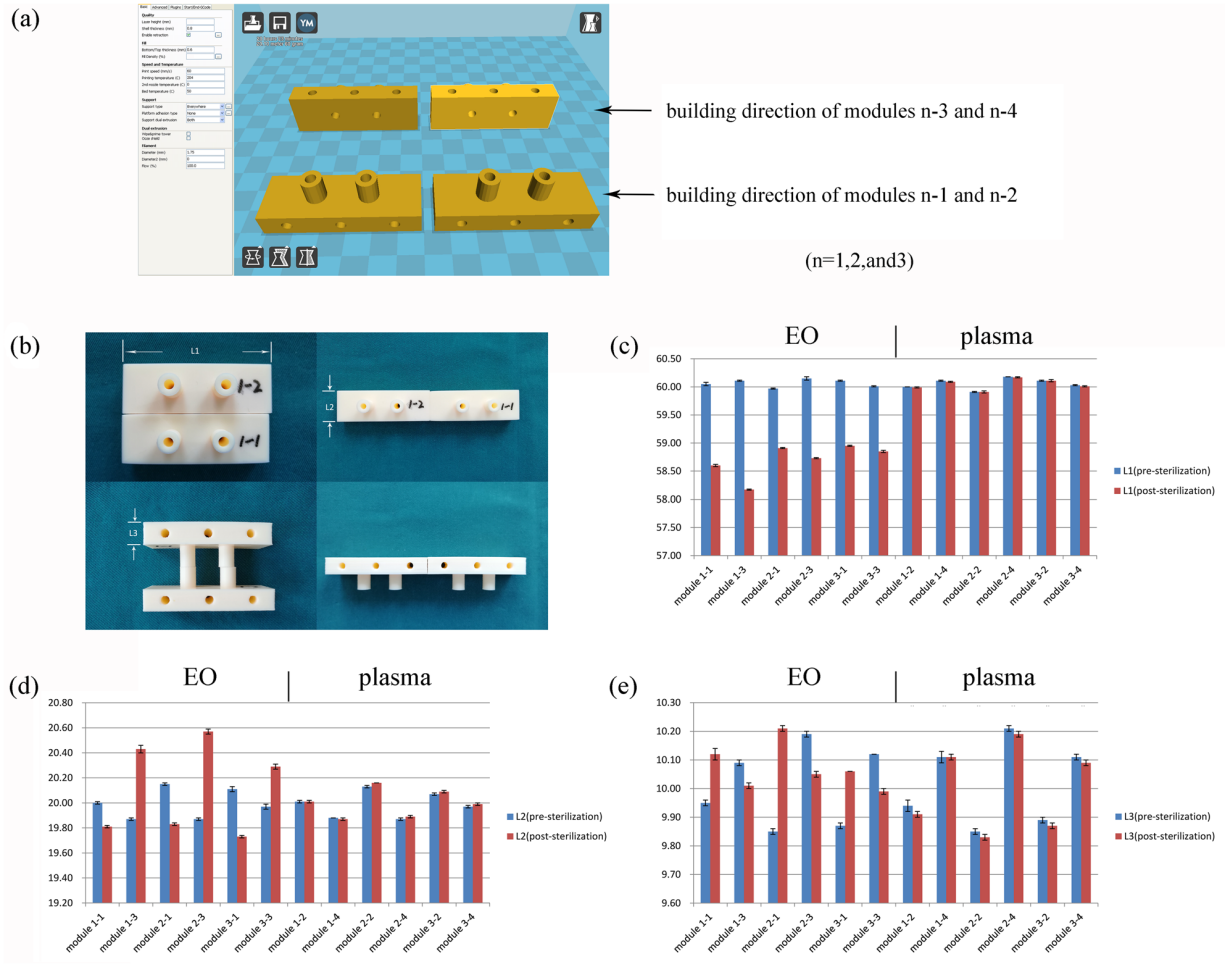
The influence of two sterilisation methods and two building directions on module deformation: (**a**) two-slicing direction was formed in Cura software, (**b**) the difference in module deformation can be observed after two kinds of low-temperature sterilisation. According to the diagram, (**c**) L1 of all modules becomes shorter after EO sterilisation, (**d, e**) L2 and L3 may be shortened or extended after EO sterilisation, and they were always extended when the building direction of L2 or L3 was parallel to the Z-axis. All experiments of every module were performed by 5 measuring points.

Modules 1–1, 1–3, 2–1, 2–3, 3–1, and 3–3 underwent EO sterilisation. In this detail, it can be seen that modules with different building directions and different infill densities eventually undergo deformation after EO sterilisation. Figure 2b shows that the modules lengthened in the Z direction after sterilisation, while they contracted in the X- and Y-directions. Modules 1–2, 1–4, 2–2, 2–4, 3–2, and 3–4 underwent plasma sterilisation, but there was no deformation before and after sterilisation, see Figs. 2c–e.

### The correlation between printing parameters of modules and micro-deformation of modules

**Figure 3:**
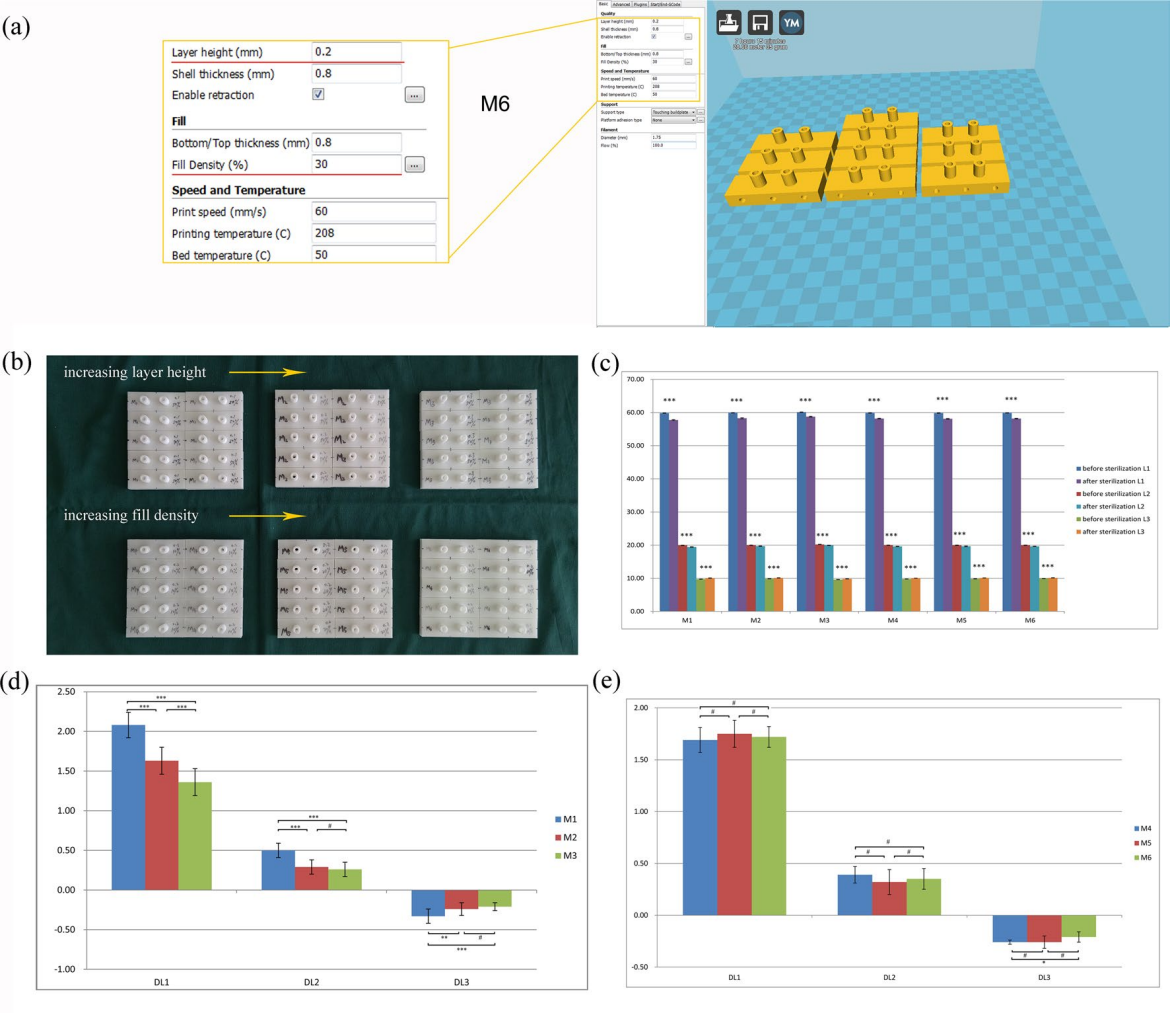
Changes of M series modules before and after EO sterilisation: (**a**) settings of the Cura software, only layer height and infill density were changed; (**b**) measuring points and parameter settings of the M series; (**c**) There are significant statistical differences in the dimension of each module before and after sterilisation; (**d**) analysis of the differences in dimensional changes between M1, M2, and M3 modules after sterilisation based on layer height; (**e**) analysis of the differences in dimensional changes between M4, M5, and M6 modules after sterilisation based on infill density. Values are the means ± SD, n = 10. ^#^*p* > 0.05, *p < 0.05, **p < 0.01, and ***p < 0.001 suggests a statistically significant difference between the indicated groups. DLn = Ln (pre-sterilisation)-Ln(post-sterilisation), n = 1, 2, and 3.

There were significant statistical differences in the dimension (L1, L2, and L3) of each module before and after EO sterilisation (*p* < 0.001, M1–M6). The shortening of L1 and L2 and the lengthening of L3 were more obvious in the module of smaller layer height (*p* < 0.001). Figure 3 shows that the difference in printing parameters affects the magnitude of deformation. Our results demonstrated that the smaller the layer height of the module, the greater the deformation of the module. The infill density of the module has a low correlation with the magnitude of deformation (*p* > 0.05). For paired *t*-test of Fig. 3, the total sample size of 10 (per group) revealed 0.80 power to detect a significant association (*α* < 0.05)with an effect size index of 1. For ANOVA of Fig. 3, the total sample size of 30 revealed 0.8 power to detect a significant association (*α* < 0.05) with an effect size index of 0.6.

### Time–temperature characteristics of M series modules

**Figure 4:**
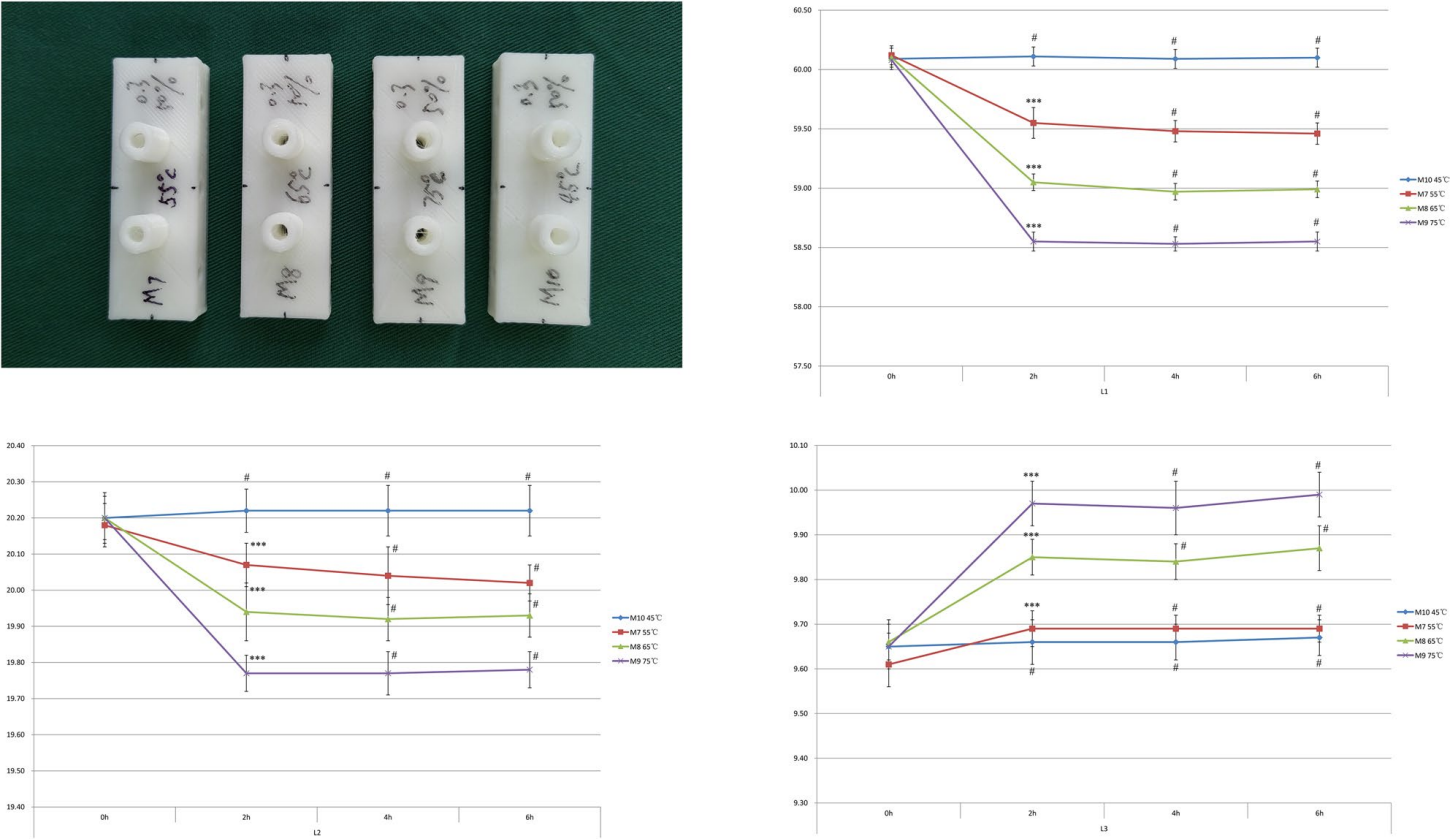
Time–temperature characteristics of M series modules. The characteristic curve shows that no deformation occurs at 45 °C, and deformation starts to occur above 55 °C. The change is most dramatic in the first 2 h. Values are the means ± SD, n = 10, ^#^*p* > 0.05, *p < 0.05, **p < 0.01, and ***p < 0.001 versus previous time point of the indicated module.

The characteristic curve showed that the deformation had not occurred at 45 °C (*p* > 0.05, M10), and the deformation began to occur above 55 °C (*p* < 0.05, M7–M9). As the temperature rose, the degree of deformation increased. The change was most dramatic in the first 2 h and negligible after that (Fig. 4). The present sample size of 10 (per group) revealed 0.80 power to detect a significant association (*α* < 0.05) with an effect size index of 1.0.

### Characterisation of PSNT using three materials and three sterilisation methods

**Figure 5:**
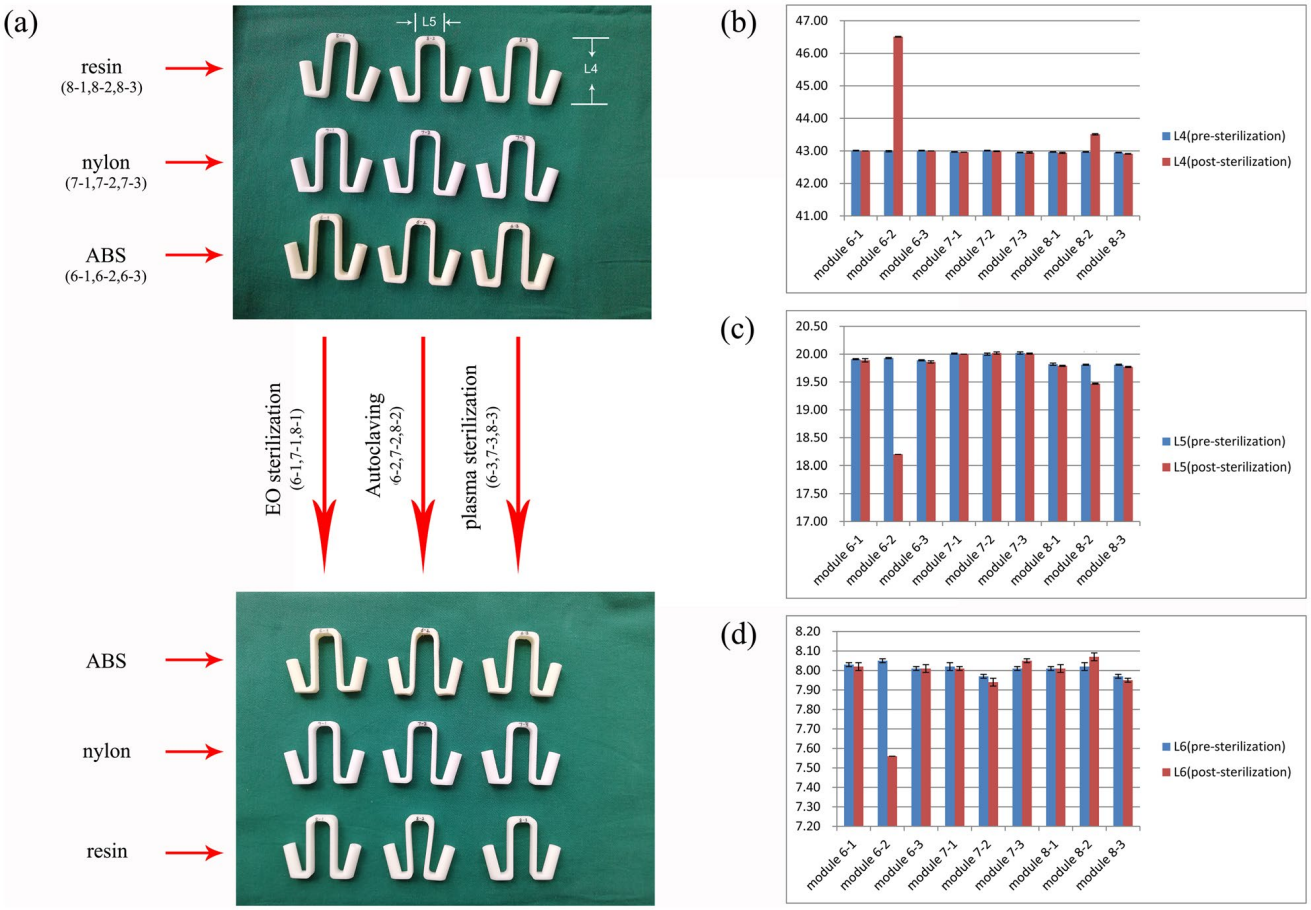
(**a**) Characterisation of PSNTs using three materials and three sterilisation methods. (**b**–**d)** there is no significant difference in PSNT deformation of the three materials before and after low-temperature sterilisation, while the PSNT of ABS and resin materials deformed after autoclaving. Among them, the deformation of ABS material is the most obvious. All experiments of every module were performed by 5 measuring points.

The results of the experiments found clear support for the template deformation characteristics being closely related to the build direction. The templates which underwent EO sterilisation and plasma sterilisation were not deformed. However, high-pressure steam sterilisation caused the ABS material printed using the FDM method and the photosensitive resin material printed using an SLA method to exhibit serious deformation. The results were consistent with the previous experiment; the templates were elongated in the Z direction and contracted in the X and Y directions. The nylon material sterilised by autoclaving showed no deformation. The six templates undergoing EO sterilisation and plasma sterilisation showed no significant changes in dimension before and after sterilisation (Fig. 5).

### Characterisation of five groups of modules before and after EO sterilisation

**Figure 6:**
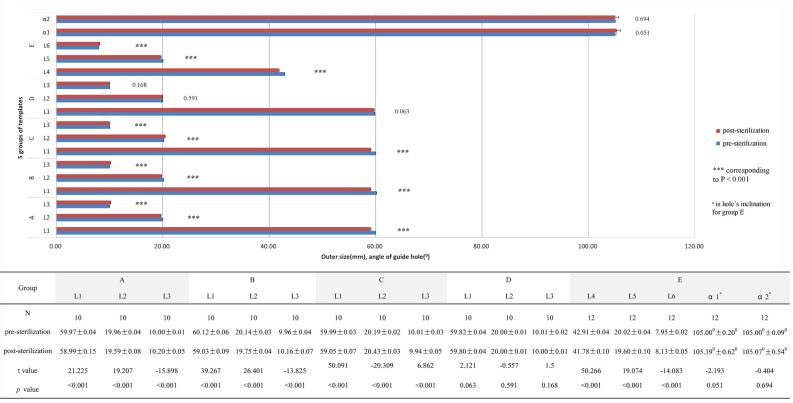
Comparison of module outer dimension for the five groups before and after EO sterilisation.

**Figure 7:**
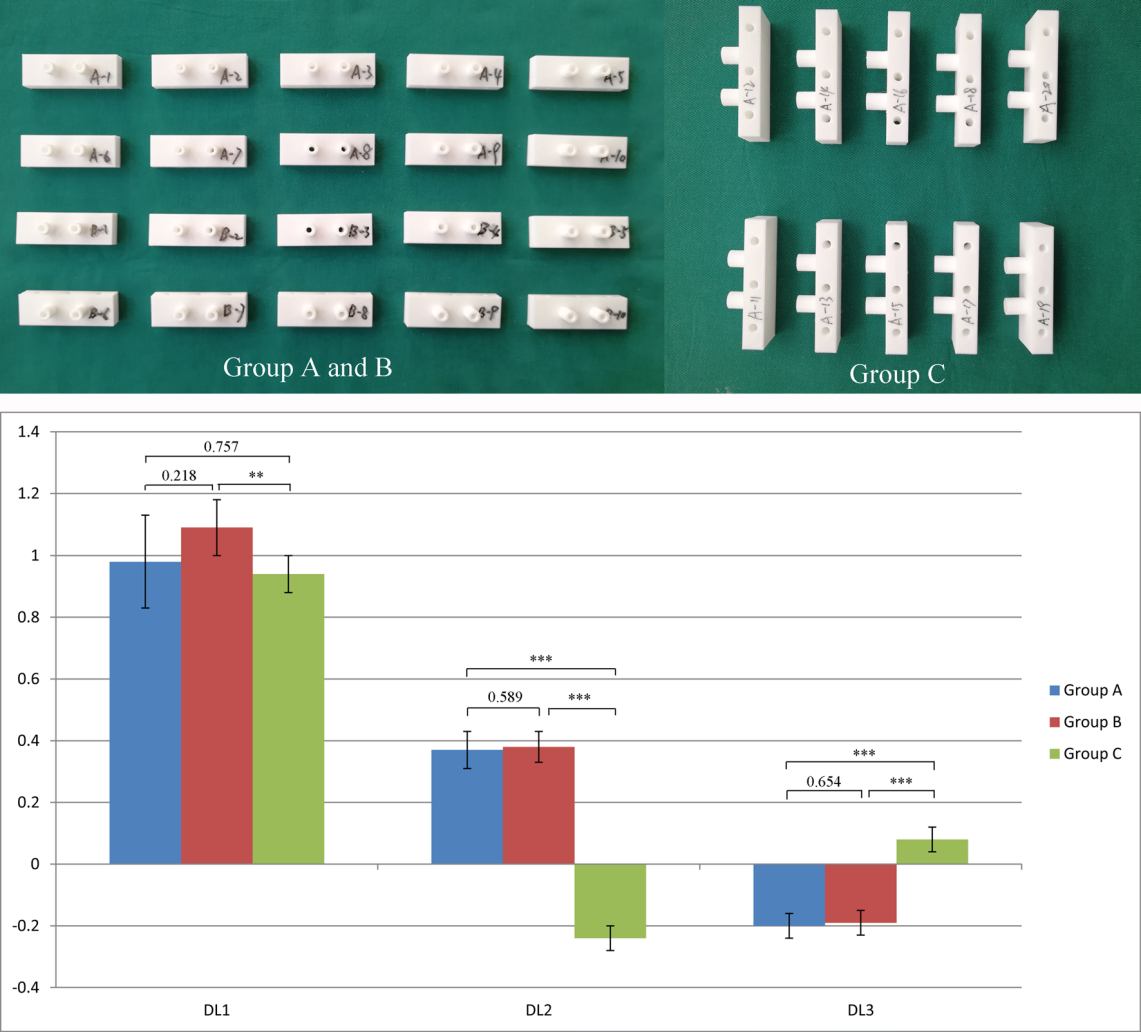
Before and after EO sterilisation, the deformation characteristics of groups A, B, and C were related to the build direction. The build direction of group A and group B were the same, and the deformation was also consistent. The build direction of group C was perpendicular to the build direction of group A and B, and the dimension changes of L2 and L3 were opposite. Values are the means ± SD, n = 10. **p < 0.01, and ***p < 0.001 suggests a statistically significant difference between the indicated groups. DLn = Ln (pre-sterilisation)-Ln (post-sterilisation), n = 1, 2, and 3.

The lengths of L1, L2, and L3 for the three groups (A, B, and C) before and after EO sterilisation showed statistically significant differences (*p* < 0.001), but the lengths of L1, L2 and, L3 for the group D before and after EO sterilisation showed no significant differences (*p* = 0.063, *p* = 0.591, and *p* = 0.168) (Fig. 6). It can be seen from a comparison between the three groups (A, B, and C) after EO sterilisation, in group C and the other two groups that the changes in L2 and L3 showed a significant difference (*p* < 0.001); in groups A and B, with the same building direction, changes in L2 and L3 showed no significant difference (*p* = 0.589, *p* = 0.654) (Fig. 7). The lengths of L4, L5, and L6 for group E showed statistically significant differences before and after EO sterilisation (*p* < 0.001), while the angle of the guide hole showed no statistical significance (*p* = 0.051 and *p* = 0.694) (Fig. 6). Thus, it can be shown that the deformation of the PSNT is related to the building direction. Besides, the results also imply that the PSNT has no bending deformation. This experiment reveals some important reasons for the positioning failure of PSNTs. For Fig. 6, The present sample size of 10 (or 12) revealed 0.80 (or 0.88) power to detect a significant association (*α* < 0.05) with an effect size index of 1.0. For Fig. 7, the present total sample size of 30 revealed 0.8 power to detect a significant association (*α* < 0.05) with an effect size index of 0.6.

## Discussion

### Concerns about the clinical application of 3D printed navigational template

Following the development of 3D printing technology in the medical field, 3D printing moulds are commonly used for preoperative planning and surgical simulation^23^. 3D printed navigational templates are helpful for the accurate positioning of operations, and due to their importance, increasing attention has been paid to the accuracy of spine positioning^24,25^. In all types of reports, however, inaccurate conditions occasionally appear; this causes concern to surgeons since some areas are close to important vascular nerves and have high requirements for positional accuracy, such as a cervical vertebra. Because of the anatomical proximity of the vertebral pedicles to associated neurovascular structures, a failure in positioning may damage the vertebral artery or cervical cord with catastrophic consequences^26^.

### Factors affecting the accuracy of the navigation template

The mechanical positioning accuracy of current 3D printers is generally about 10 μm, and the precision of the navigation template itself is often associated with the parameter settings of the machine and the material properties. It is generally believed that the thinner the layer thickness, the smaller the nozzle, and the slower the printing speed, the higher the printing accuracy based on the parameters of the 3D printer. The study found that the layer thickness reaches 0.1 mm, the nozzle diameter reaches 0.25 mm, and the printing speed is set to 30 mm/s, which has reached the accuracy limit of the FDM printer. The use of a stepper motor avoids cumulative errors and gives a deviation in the 3D printed navigational template of usually no more than 0.2 mm, meaning that the precision is high enough for use in a clinic. The most commonly used materials in FDM printing include ABS and PLA, while SLA and digital light processing (DLP) printing typically uses photosensitive resin material. SLS printing is commonly used for nylon materials. For some complex structures that are prone to warping and deformation, PLA has the advantages of safety, environmental protection, and non-deformation, and is often used in model designs^27^.

The design of the navigation template should correspond to the local anatomical structure and operational area. Due to the attachments of muscles and ligaments, and the presence of important blood vessels and nerves, some areas cannot be shown, meaning that some templates are difficult to fit. The existence of cartilage is another factor that affects the positional precision of the navigation template. In elderly patients, due to osteoporosis, the cavity of bones model is common. Unless a "fill" operation is conducted on the surface of the model in advance, it will be hard to attach the template to the bone surface^28^. Besides, when these navigation template designs are thinner, the guide hole will be deformed by the surgeon's gestures during operation, resulting in a change in the orientation angle. The fit clearance of the guide hole and the tool will also cause angular deviation. Some designs for PSNT involve two segments, which is feasible for ankylosing spondylitis, but for patients with spinal degeneration, due to the supine position used when acquiring CT data and the prone position used in the operation, the difference in the curvature of the spine will lead to deviation in the navigation template and a positioning error. The weird thing is that well-designed PSNTs have good accuracy in a simulation operation of the spine model, but a positioning holes deviation is found when applied to the real operation.

Originally, it was believed that this was caused by bad treatment for the bone surface, shifting of the navigation template, or poor design. Following a review of each detail, we focus here on the sterilisation process. Most of the polymeric biomaterials have relatively low melting temperatures and are susceptible to degradation and/or morphological degeneration at high temperatures. According to Savaris^29^, the sterilisation process by EO, hydrogen peroxide plasma, electron beam radiation, and gamma radiation can be used for PLA sterilisation. However, our test results show that EO sterilisation still causes deformation and the layer-by-layer processing method involved in 3D printing is related to the deformation characteristic of the material. A similar pattern of results was obtained in Fig. 6.

### Sterilisation correlation

Although the *T*_*m*_ of PLA is about 200 °C, experiments show that it undergoes deformation following low-temperature sterilisation at about 55 °C, and this is related to the properties of the material. This type of polymer undergoes a transition at a certain temperature before it is converted into the fluid; this is known as the glass transition temperature (*T*_*g*_), the temperature at which a polymer material is converted from glassy to an elastomeric state and is an important physical property of a non-crystalline polymer. For PLA, the *T*_*g*_ decreased after any of the sterilisation methods were employed^16^. This could explain the micro-deformation of PLA materials after EO sterilisation. The *T*_*g*_ for PLA and HT-PLA are only 55 °C, which is the root cause of the deformation appearing after EO sterilisation. The *T*_*m*_ for the ABS material used for 3D printing is 260 °C, although serious deformation is shown under high-pressure steam sterilisation at 121 °C since the *T*_*g*_ is about 90 °C. The FDM forming method weakens the macroscopic surface adhesion, and its deformation involves stretching of the build direction. Some 3D printing materials contain added plasticisers to change product performance, which leads to a decrease in *T*_*g*_, negatively affecting the sterilisation of the navigation template.

According to the manufacturer's data, the *T*_*g*_ of the materials used in the experiments were 55 °C for PLA and HT-PLA, 86 °C for ABS, 67 °C for high-temperature photosensitive resin, and 120 °C for nylon. Setting the EO sterilisation temperature to 55 °C, plasma sterilisation temperature to 48–55 °C, and high-pressure steam sterilisation temperature to 134 °C, our results demonstrated that chemical-based sterilisation could still cause the deformation of materials with low *T*_*g*_. Although the *T*_*g*_ of the PLA material is equivalent to the EO sterilisation temperature and the plasma sterilisation temperature, the experimental results showed that the PLA materials which underwent EO sterilisation were deformed, and those which underwent plasma sterilisation were not deformed (Fig. 2). This may be related to the sterilisation time and gas. HR-PLA has the same *T*_*m*_ as PLA, but its *T*_*g*_ is nearly 10 °C higher, so there was no obvious deformation in chemical-based sterilisation (Fig. 6). The present study confirmed that the deformation of the material depends on its *T*_*g*_, sterilisation temperature, and duration of sterilisation cycle. Therefore, for intraoperative applications of the navigation template, materials made under different processing conditions still need to undergo a deformation test.

To avoid the interference of chemical-based sterilisation, we experimented with a constant temperature drying oven and drew the time–temperature curve of HT-PLA. Figure 4 shows that the deformation starts at 55 °C, which is close to the *T*_*g*_ of HT-PLA. From the time–temperature curve, it is clear that the main deformation period of the module is concentrated within 2 h. On this basis, we note that inappropriate ambient temperature will affect the accuracy of the product when using 3D printed products.

### Deformation characterisation

Compared with SM, the mechanical properties of products produced using AM are poor, and the main reason for this is that a layered manufacturing process causes anisotropy in the mechanical properties^30,31^. Therefore, the thermodynamic deformation response of a 3D-printed template involves anisotropy. The inevitable lower density in the Z direction means that it is elongated.

The strength of FDM parts across the layers (in the Z direction) is significantly lower than the strength along the threads (X and Y direction), sometimes by an order of magnitude. The most important factor that defines the strength of the resulting part is the interlayer contact surface area, which is defined by nozzle diameter to layer thickness ratio. The part strength decreases along with the layer thickness increase^32^. The influence of different printing parameters on the degree of deformation is shown in Fig. 3. Results indicate that the layer height is inversely proportional to the degree of deformation. It is generally believed that the deformation will be severe after heating due to the worse mechanical properties when the layer height increases. However, experiments have found that the smaller the layer height, the greater the degree of deformation. Unfortunately, the reason is not clear. We also found that the strength of HT-PLA was higher than that of PLA, but its deformation was not improved in low-temperature sterilisation. It is worth mentioning that the deformation of the template always follows the anisotropic characteristics, although the printing parameters change.

The SLA 3D printed product has both the best quality and the highest precision. SLS printed products have a frosted texture. The advantages of this method are that it can print using metal or nylon, and the strength and heat resistance of these templates are superior to those using the other two types of 3D printing. FDM printed products have the lowest cost and lower accuracy. Figure 5 used 3 kinds of materials and 3 kinds of sterilisation methods for the experiment; the results are obvious, the *T*_*g*_ of ABS, photosensitive resin, and nylon is higher than the chemical-based sterilisation temperature so there was no deformation. The high-pressure steam sterilisation temperature is higher than the *T*_*g*_ of ABS and photosensitive resin, therefore deformation is inevitable. Sharma found that steam sterilisation resulted in an overall linear expansion of the photopolymer resin material, with an increase in outer dimensions and a decrease in inner dimensions^21^. However, we found the deformation law that the post-sterilisation module obeys: The module stretches in the Z direction and shrinks in the X and Y directions.

Analysing the data, we found that the deformation of the module using low-temperature sterilisation was only about 1 mm (L1 in Fig. 6), which has little effect on some low-demand surgical guides (such as knee joint osteotomy). However, for high-precision surgery, risks will be difficult to avoid (such as cervical spine PSNT). Therefore, a correct understanding of the micro-deformation of surgical guide is essential for clinical surgery.

**Figure 8:**
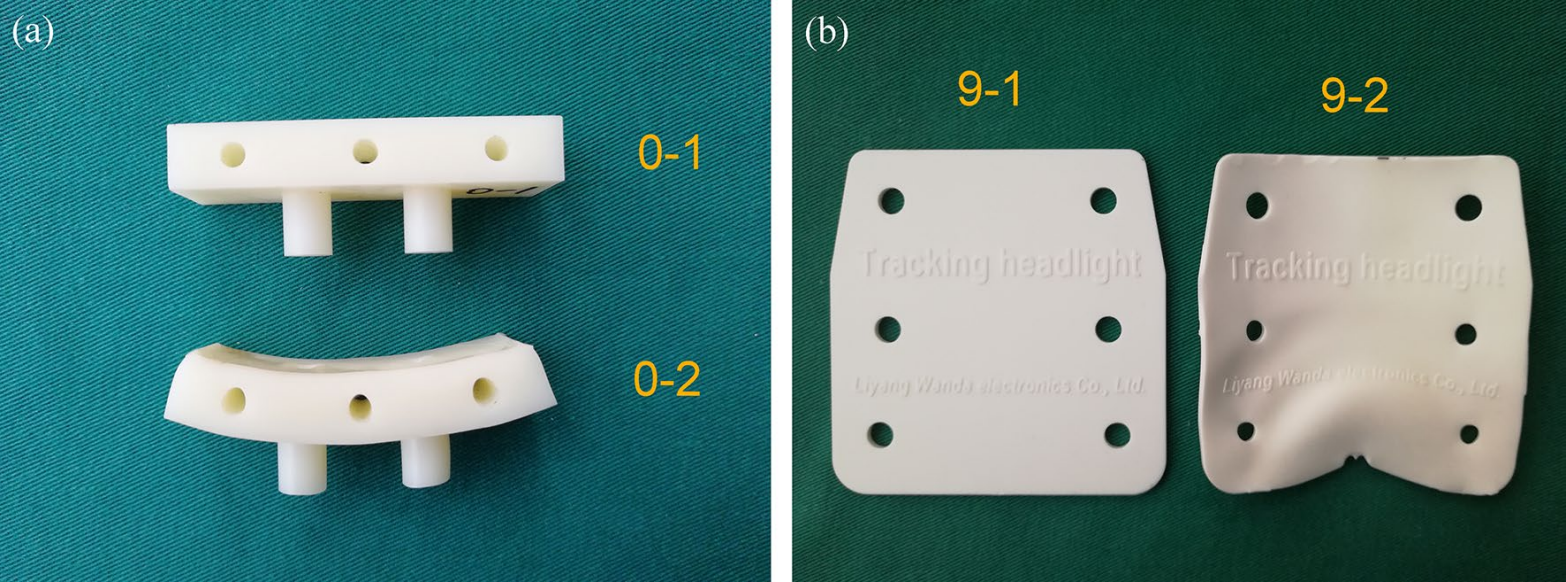
Deformation characteristics of SM products: (**a**) the CNC machined module showed no change after EO sterilisation (0–1), while severe deformation appeared after autoclaving (0–2); (**b**) the injection moulded product showed no change after EO sterilisation (9–1), while severe deformation appeared after autoclaving (9–2).

For SM processing, we chose CNC and injection moulding. Both methods have excellent machining accuracy and fine surface effects. Figure 8 shows that the two kinds of SM processed products have undergone significant deformation after autoclaving. The deformation lacks regularity. This is related to the isotropy of the SM processed products.

### Solutions and clinical applications

**Figure 9:**
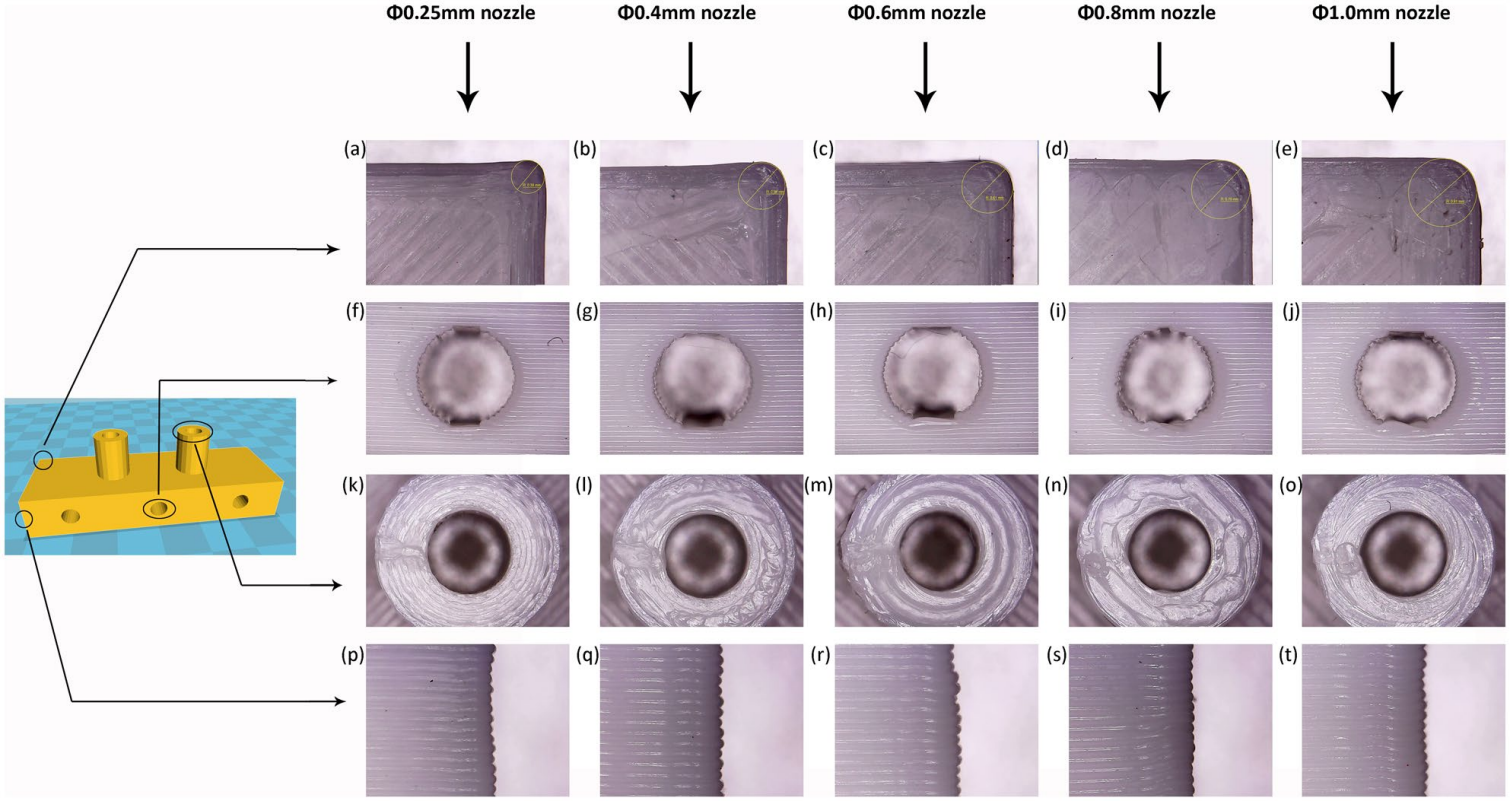
The influence of the nozzle diameter on the dimensional accuracy of the module. As the nozzle diameter increases, (**a–e**) the tip corners of the modules become more blunt, (**f–j**) the quality of the holes parallel to the x–y axis decreases, (**k–o**) the quality of the holes parallel to the z-axis is consistent, and (**p**–**t**) the surface quality of the module is also consistent.

When the navigation template is used in the clinic, there are two key parameters, the strength of the part and dimensional accuracy, that need to be considered. Z-direction strength weakness has been attributed to poor bonding between printed layers. This bonding depends on the *T*_*m*_ of the current layer being deposited and the temperature of the previously deposited layer. When the depositions of filaments bring the temperature of the filaments to a temperature greater or equal to the crystallisation temperature (*T*_*c*_), the inter-filaments bonding strength will undergo an additional improvement^33^, but surface re-flow causes damage and affects the quality of printed parts because of pre-heating^34^. In other words, increasing the bed temperature can improve the adhesion between the printed layers, but it will affect the accuracy of the guide plate. Part qualities are greatly influenced by the various process parameters, layer thickness, layer width, and infill orientation/density play a critical role in the mechanical properties of the part produced^35^. The infill density is the most significant parameter determining the tensile strength and elastic modulus of the printed part. The infill pattern also affects the mechanical properties of the part. Triangular, grid and hexagonal infilled parts resulted in similar ultimate tensile strength, the concentric pattern was not recommended for torsional applications, because due to its symmetrical geometry the torsional stiffness will fall. The nozzle diameter also affects the mechanical properties of the parts produced by FDM. At constant layer thickness, an increase of nozzle diameter resulted in higher flexural strength, increasing the nozzle diameter can improve the bonding strength between printed layers. Due to the change in the volume of the output filament, the material can better adhere to the bed with a large-diameter nozzle, and the printing speed is faster. The quality can still be guaranteed for printed parts without sharp structures. The disadvantage is that the corners of the printed module are not sharp enough, and the subtle parts of the module are printed rough. To further confirm, Five kinds of the nozzle were used, with a infill density of 20% and a layer height of 0.2 mm, modules were printed separately and compared the local details (Fig. 9). Our study concluded that, for navigation templates, to ensure its accuracy and strength, it is not suitable to use a nozzle with a diameter larger than 0.4 mm and set a parameter with a layer height greater than 0.2 mm.

After long-term practice, we have found that some software operations, such as “Smoothing”, “Wrap”, and “Triangle Reduction”, are not suitable for the navigation template, because it will affect its fineness and conformability. The design of the navigation template is very important, which requires the researcher to be very familiar with the local anatomy and surgical exposure area, except for the loss of accuracy due to parameter selection, material selection, or the accuracy of the STL file. In the design process of the navigation template, only the DICOM data imported by CT is available. During the operation, some ligaments and cartilage components cannot be removed. This needs to be considered in the navigation template design process to keep about a 1 mm gap. Due to the threshold, some osteoporosis areas may be lost, which seriously affects the design accuracy. It is evident that, for clinicians, the placement stability and space tolerance of the navigation template is the most important, which is related to whether it can be used during surgery. Factors like humidity, material properties, and environment have also effects on the printing process. Therefore, the printing module is recommended to be stored indoors and used in a short.

PLA is favoured for its biodegradability, absence of unpleasant odours when heated, its overall environmental compatibility in all aspects of its life cycle, and its minimum level of residual stresses in the printed parts. Additionally, PLA emits ten times less potentially dangerous ultra-fine particles than ABS^32^. Experimental tests have shown that PLA will deform after EO sterilisation, although it does not deform after plasma sterilisation. ABS and the photosensitive resin material can be sterilised at low temperatures, including EO and plasma sterilisation, with good stability. Now, some new processes have been able to modify the PLA material to increase its *T*_*g*_^36,37^, so that a variety of sterilisation methods can be used to sterilise the navigation template without deformation. The module produced using pure white PA2200 nylon via the SLS or FDM method underwent no obvious changes in dimension after chemical-based sterilisation and autoclaving and meets EN ISO 10993-1 and USP/level VI/121°C standards for biological compatibility. PEEK (Polyetheretherketone) is one of the widely used high-performance polymers, and the tensile strength of PEEK is noticeably higher than PLA, ABS, and Nylon^30^. PEEK materials have *T*_*g*_ up to 143 °C and can be well stabilised using a variety of sterilisation methods. Referring to the EO steriliser operation manual, EO sterilisation also has a low temperature “cool” mode, which is only 37 °C and is suitable for ordinary PLA materials. Unfortunately, the ventilation time requires 32 h, which seriously affects the efficiency of clinical work. Therefore, EO sterilisation rarely uses the “cool” mode. The shortcoming of this study is that the use of vernier calipers to measure the dimension of the module. Although repeated, single-person, and fixed-point measurements were used, vernier calipers have various measurement uncertainties, for instance the accuracy of the vernier calipers, the operator variability, and thermal expansion of the parts. Finally, the measurement module aperture was abandoned.

**Figure 10:**
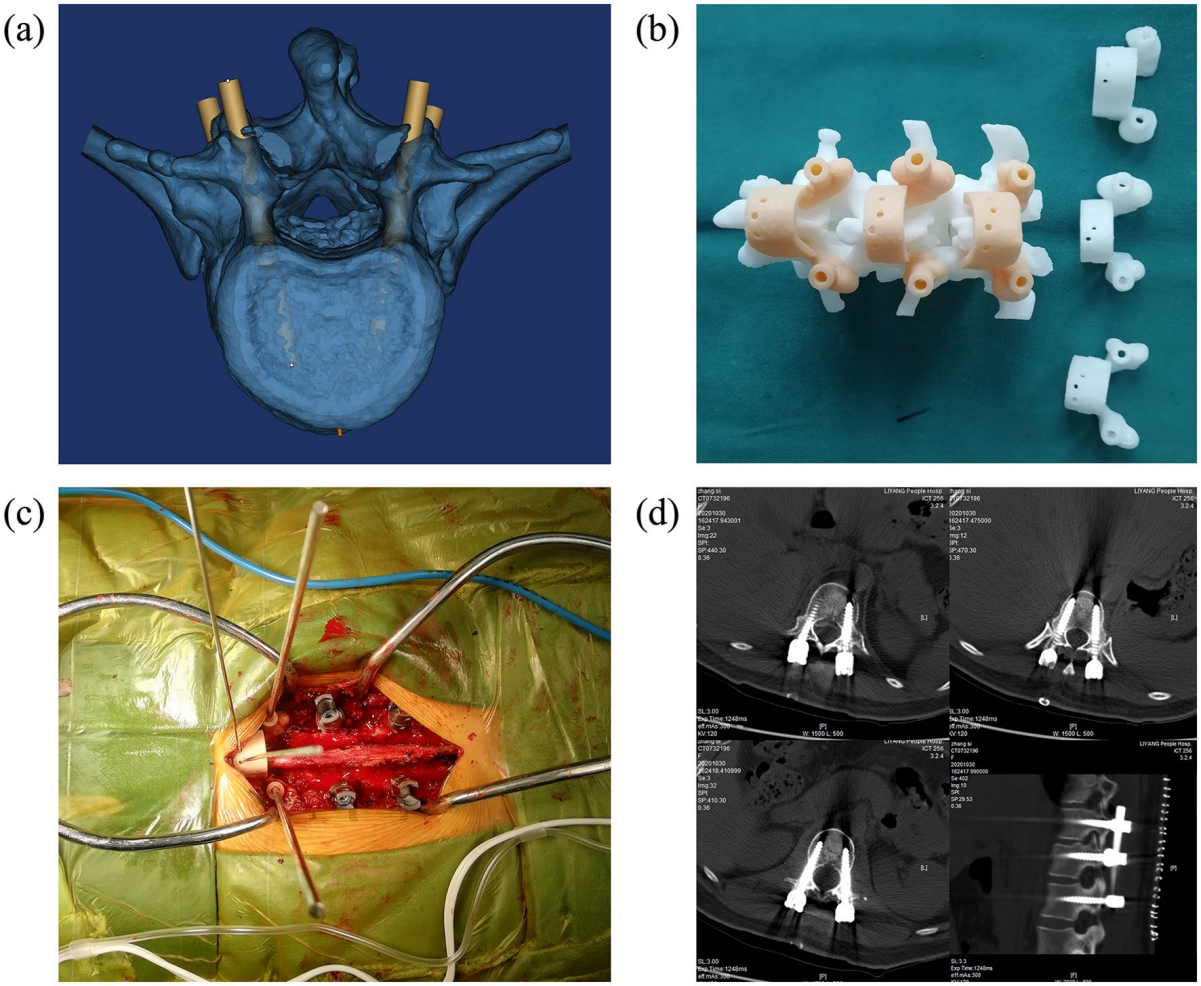
Clinical application of PSNT: (**a**) an established pedicle screw channel in Mimics software; (**b**) PSNTs were designed in 3-Matic software. Then, the PSNTs were printed with PLA and resin respectively and were sterilized by the plasma; (**c**) during the operation, the PSNTs matched the bone surface well, and the screw channel was positioned accurately; (**d**) Postoperative CT showed that the position of 6 pedicle screws was excellent.

Based on this research, we designed PSNTs, which were printed with PLA and photosensitive resin respectively. To avoid deformation of PSNTs, we adopted plasma sterilisation. The PSNTs showed a good match with the bone surface during the operation (Fig. 10).

## Conclusion

In summary, for clinical applications, the strength of the part and dimensional accuracy of the navigation template is the most important. Process parameters play a significant role in the determination of the mechanical properties of the part made by FDM. Based on ensuring dimensional accuracy, PLA materials can be sterilised by plasma, while PA and PEEK materials can be sterilised by chemical-based and autoclaving. In addition to the loss of accuracy due to parameter selection, material selection, or the accuracy of the STL file, the design of the navigation template is also very important. This paper argues that EO sterilisation will cause micro-deformation for a 3D printed navigational template made from PLA material via FDM, and these micro-deformation characteristics depend on the building direction, i.e., the module stretches in the Z direction and shrinks in the X and Y directions. These basic findings are consistent with the anisotropy of 3D printed models. The key factor causing the deformation of a 3D printing guide is its *T*_*g*_. When sterilisation conditions are the same, the layer height of the module is inversely proportional to the degree of deformation. The deformation time of the module is concentrated within 2 h. The micro-deformation of the 3D printed navigational template is regular and uniform, which leads to imperceptible risks in clinical applications.

## Supplementary Information

Supplementary Information.

## Data Availability

Data relevant to the article can be found using the Mendeley Data, V3. https://doi.org/10.17632/gw63yvcx65.3.

## Abbreviations

ABS: Acrylonitrile butadiene styrene
AM: Additive manufacturing
CNC: Computer numerical control
CT: Computer tomography
DLP: Digital light processing
EO: Ethylene oxide
FDM: Fused deposition modeling
HR-PLA: Heat-resisting polylactide
HT-PLA: High toughness polylactide
PA: Polyamide
PC: Polycarbonate
PEG: Polyethylene glycol
PLA: Polylactic
PSNT: Pedicle screw navigational template
SLA: Stereo lithography appearance
SLS: Selective laser sintering
SM: Subtractive manufacturing
STL: Stereolithography (file format)
*T*_*c*_: Crystallization temperature
*T*_*g*_: Glass transition temperature
*T*_*m*_: Melting temperature
TyrPC: Tyrosine-derived polycarbonates
PEEK: Polyetheretherketone
