# Supplementary material for: Dimensional accuracy of 3D printing navigation templates of chemical-based sterilisation
Source: Sci Rep. 2022 Jan 24;12:1253. doi: 10.1038/s41598-022-05412-7 (PMC8786919; doi:10.1038/s41598-022-05412-7)
Supplement: Supplementary file 1 — Supplementary Information. [file 41598_2022_5412_MOESM1_ESM.pdf]

Group A:

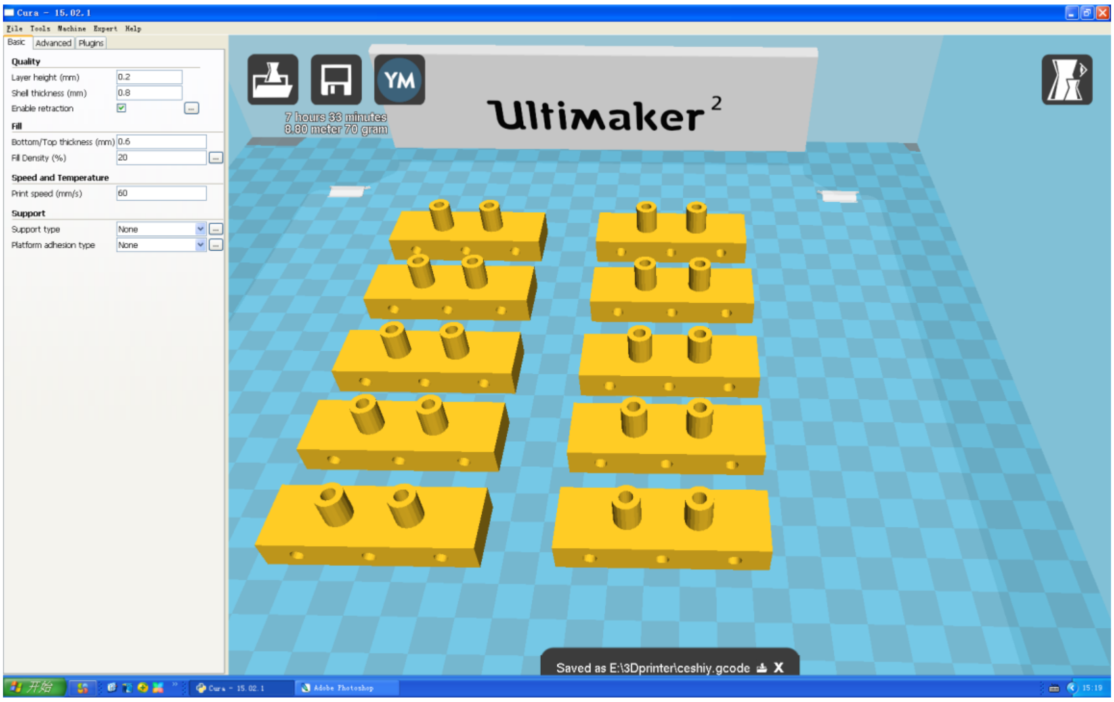

Figure S1. Slice using CURA software.

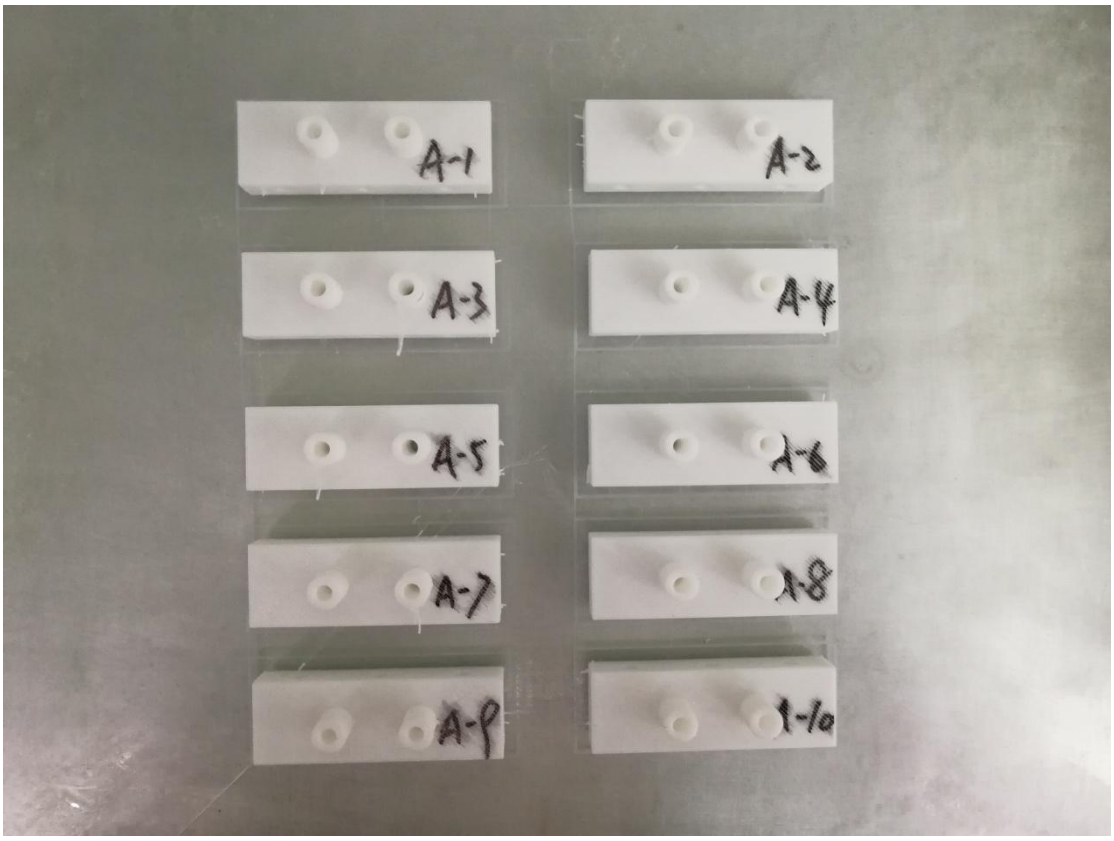

Figure S2. 3D printing was complete.

Group B:

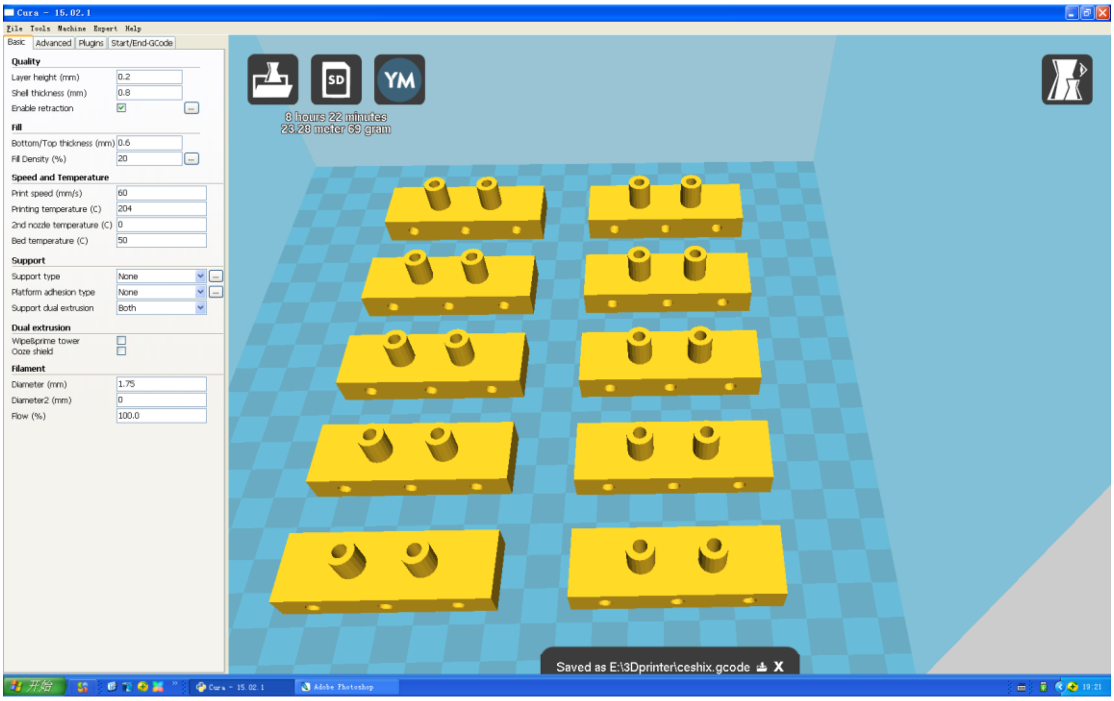

Figure S3. Slice using CURA software.

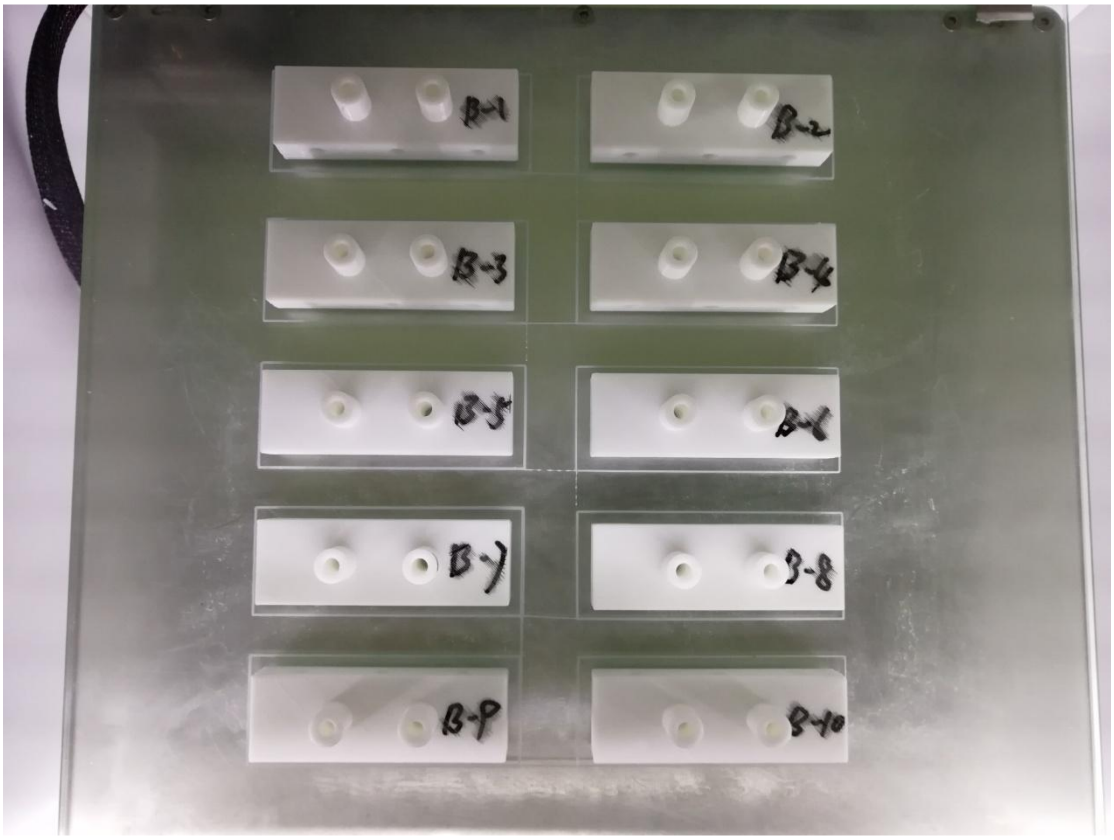

Figure S4. 3D printing was complete

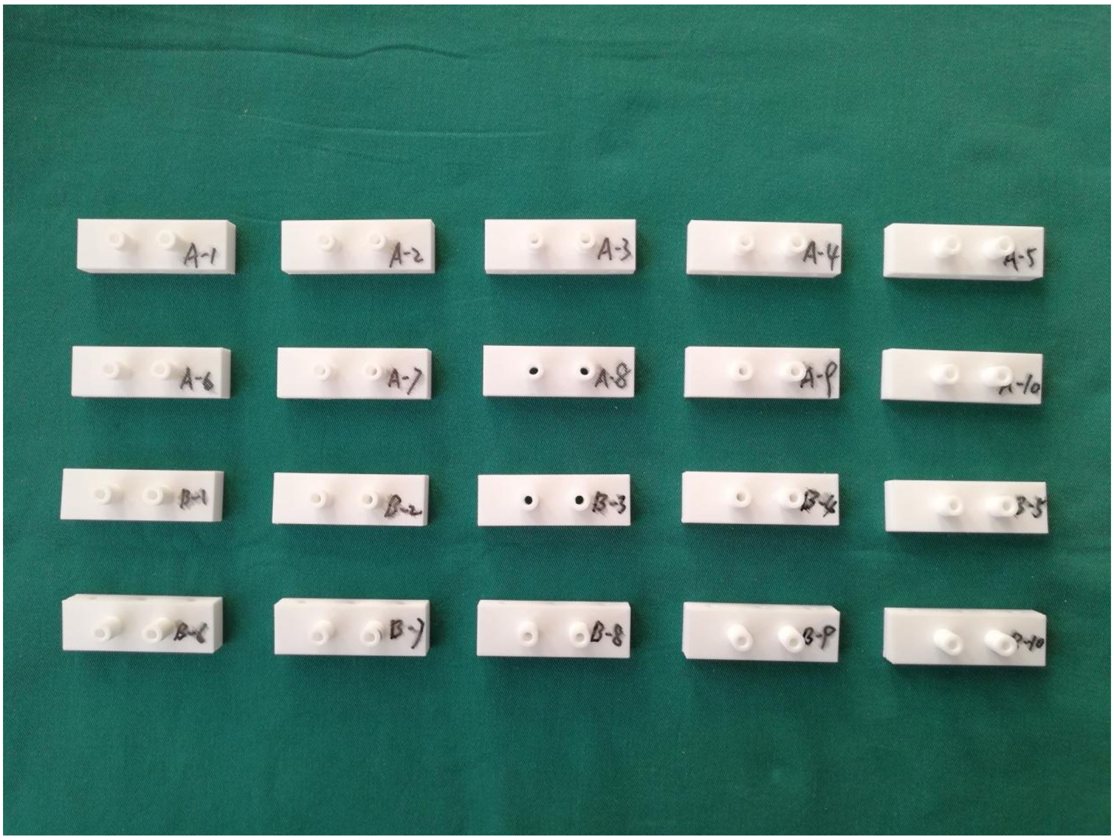

Figure S5. All modules of Group A and Group B.

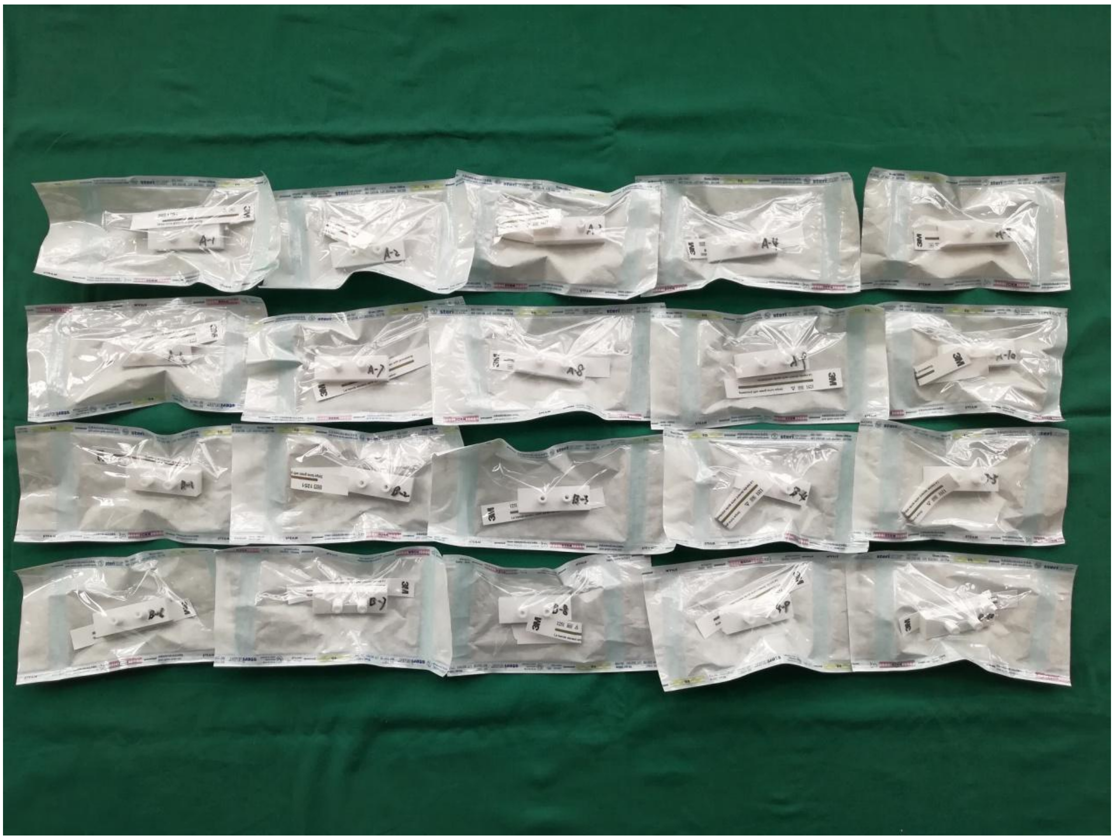

Figure S6. EO sterilization

Group C:

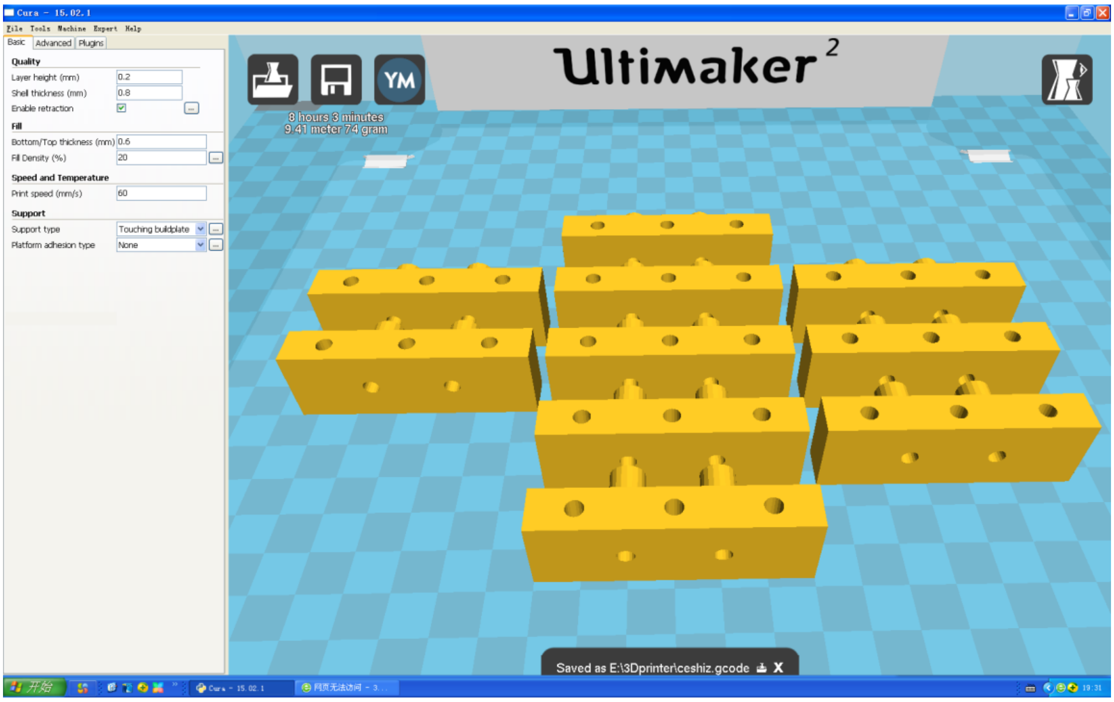

Figure S7. Slice using CURA software.

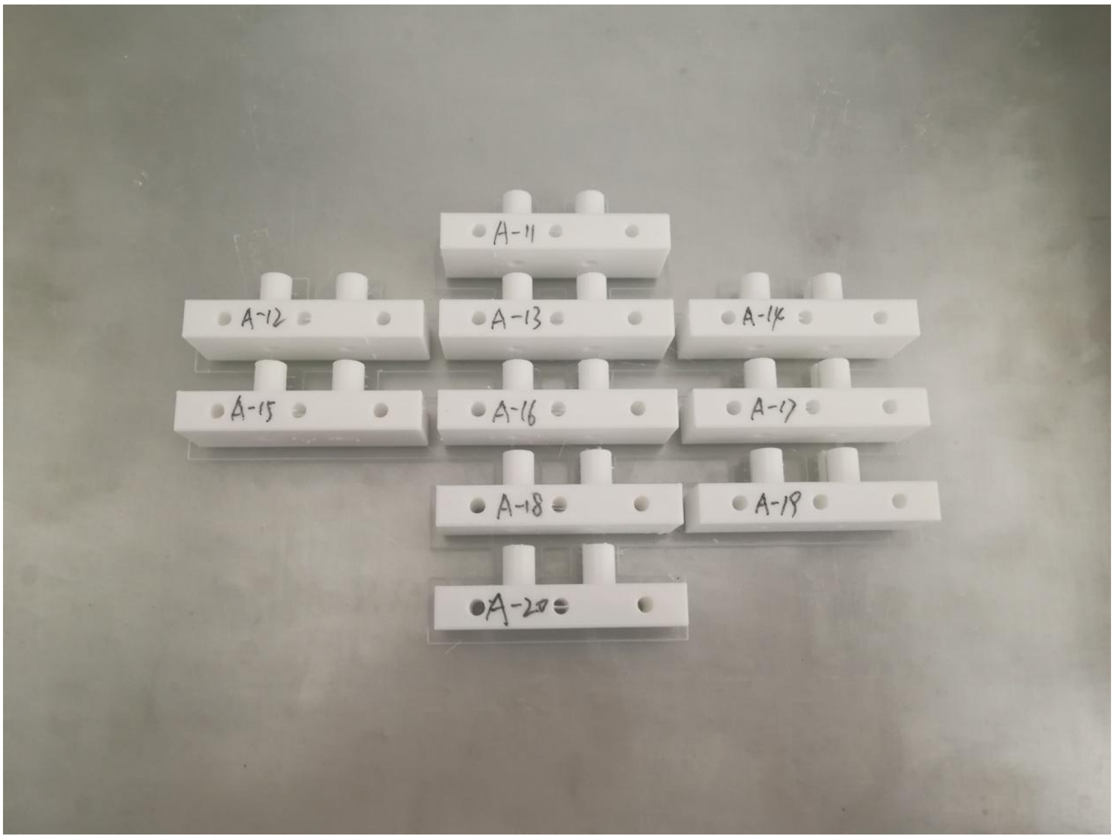

Figure S8. 3D printing was complete.

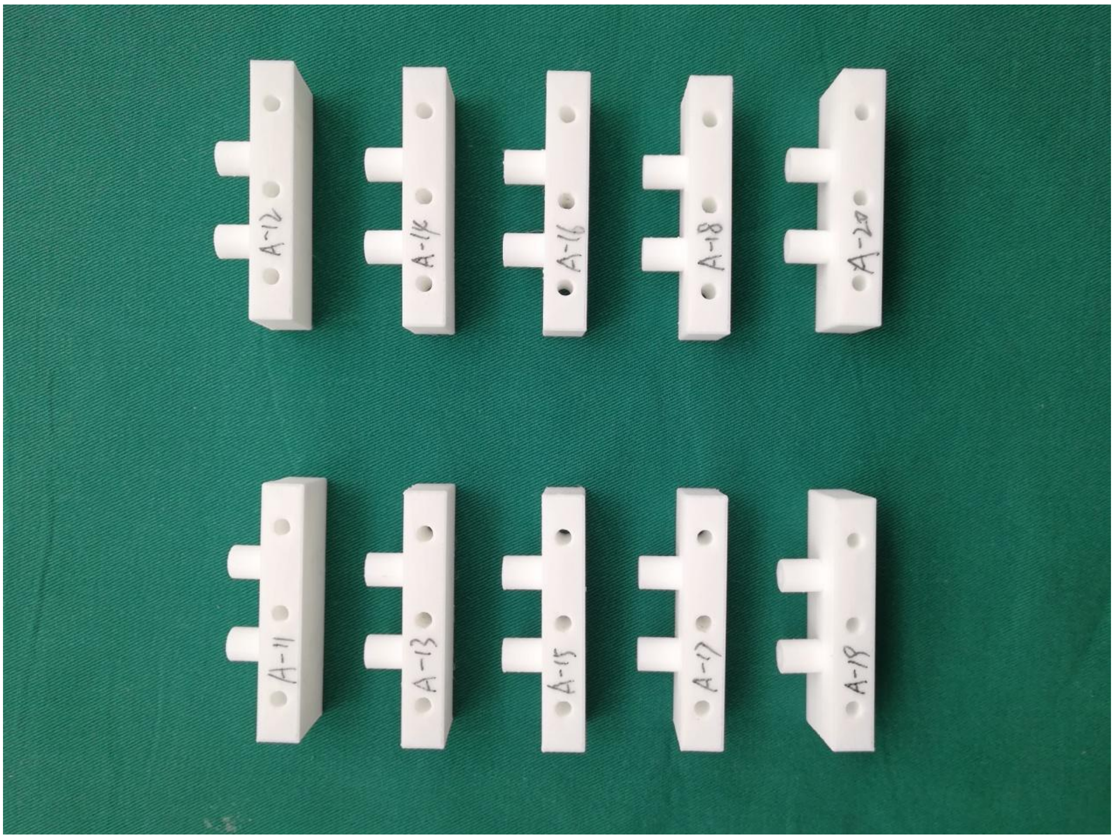

Figure S9. All modules of Group C.

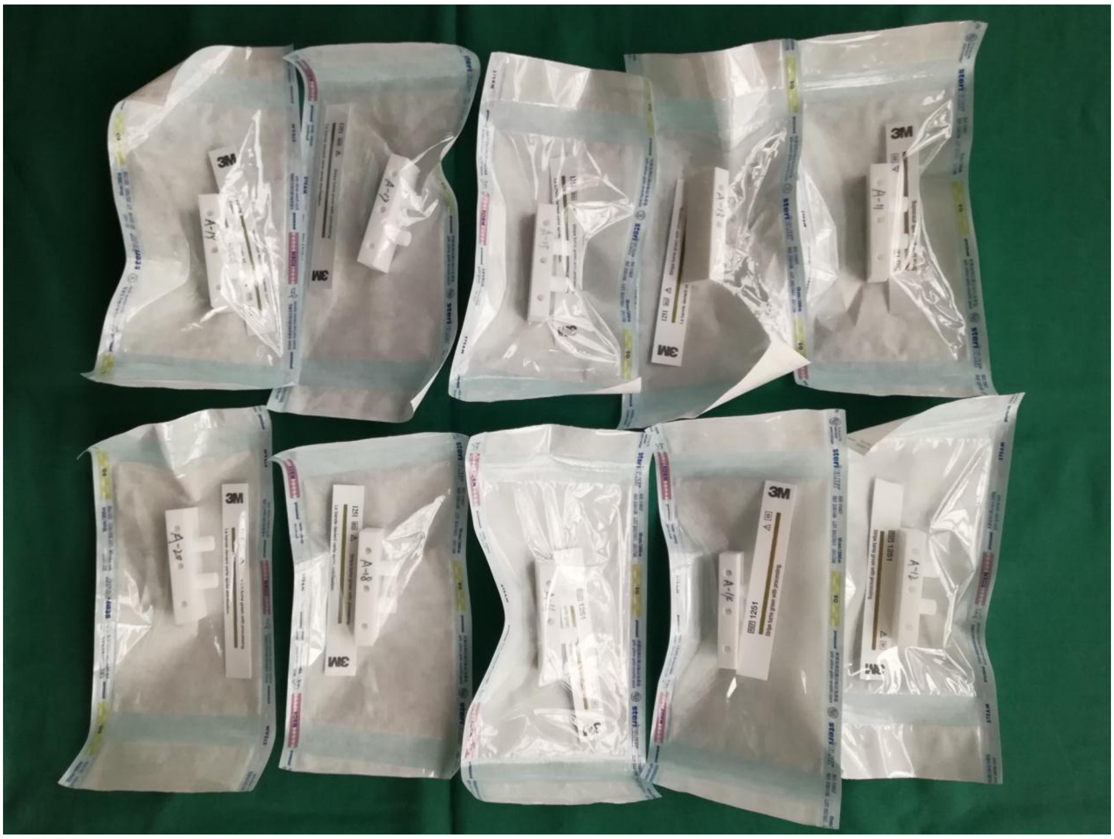

Figure S10. EO sterilization.

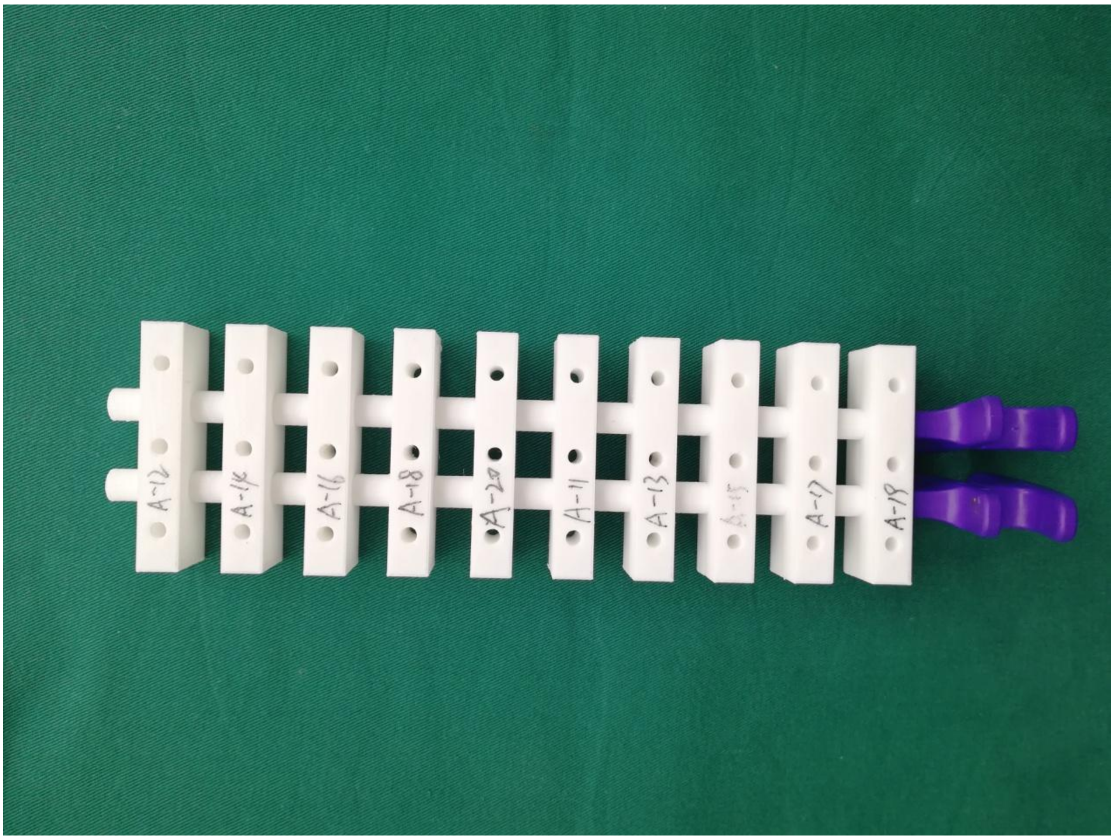

Figure S11. Positioning hole measurement.

Group D:

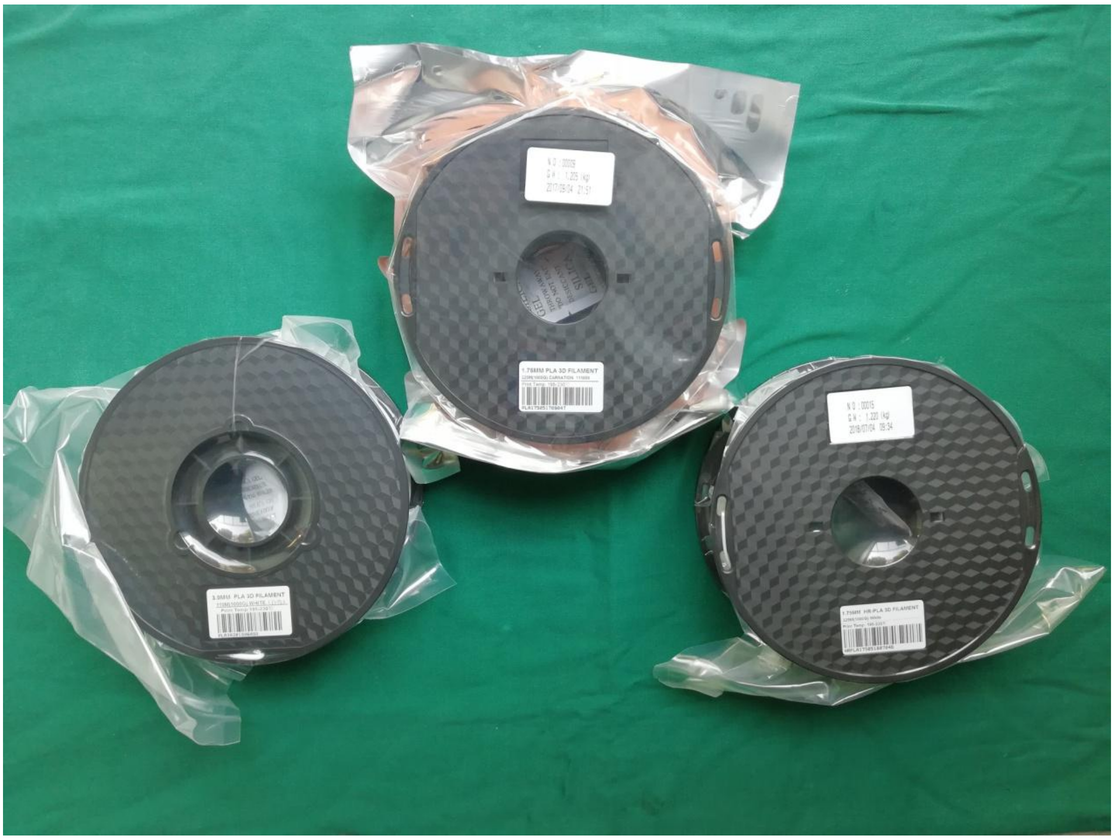

Figure S12. Consumables selection: adopt HR-PLA.

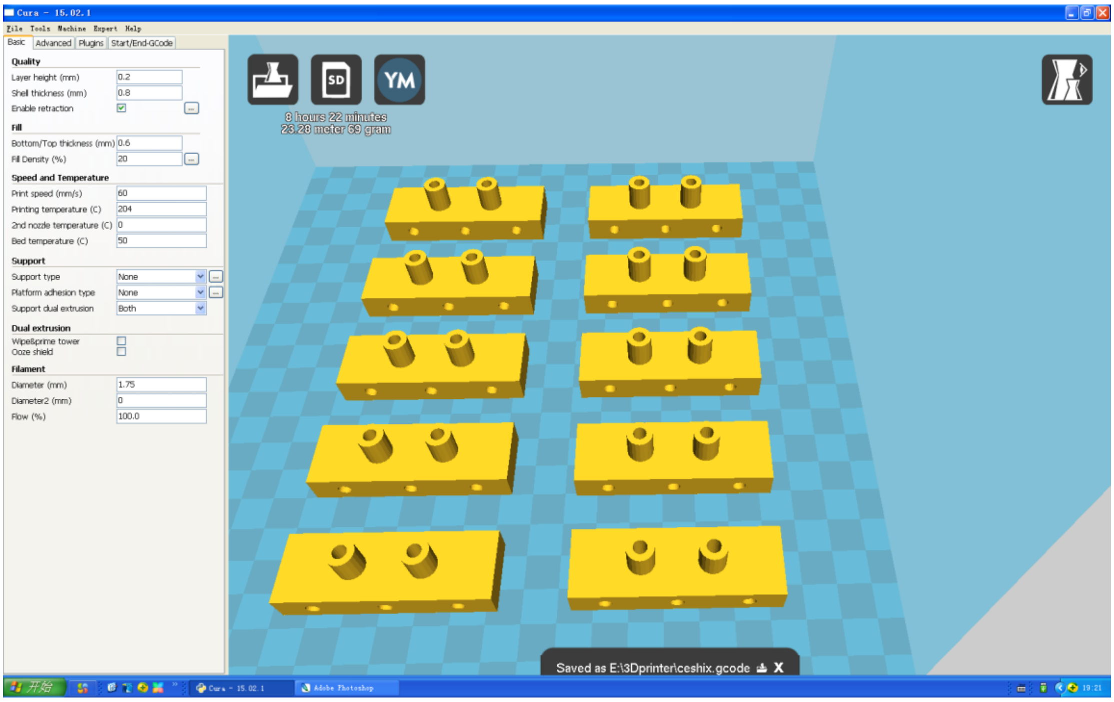

Figure S13. Slice using CURA software.

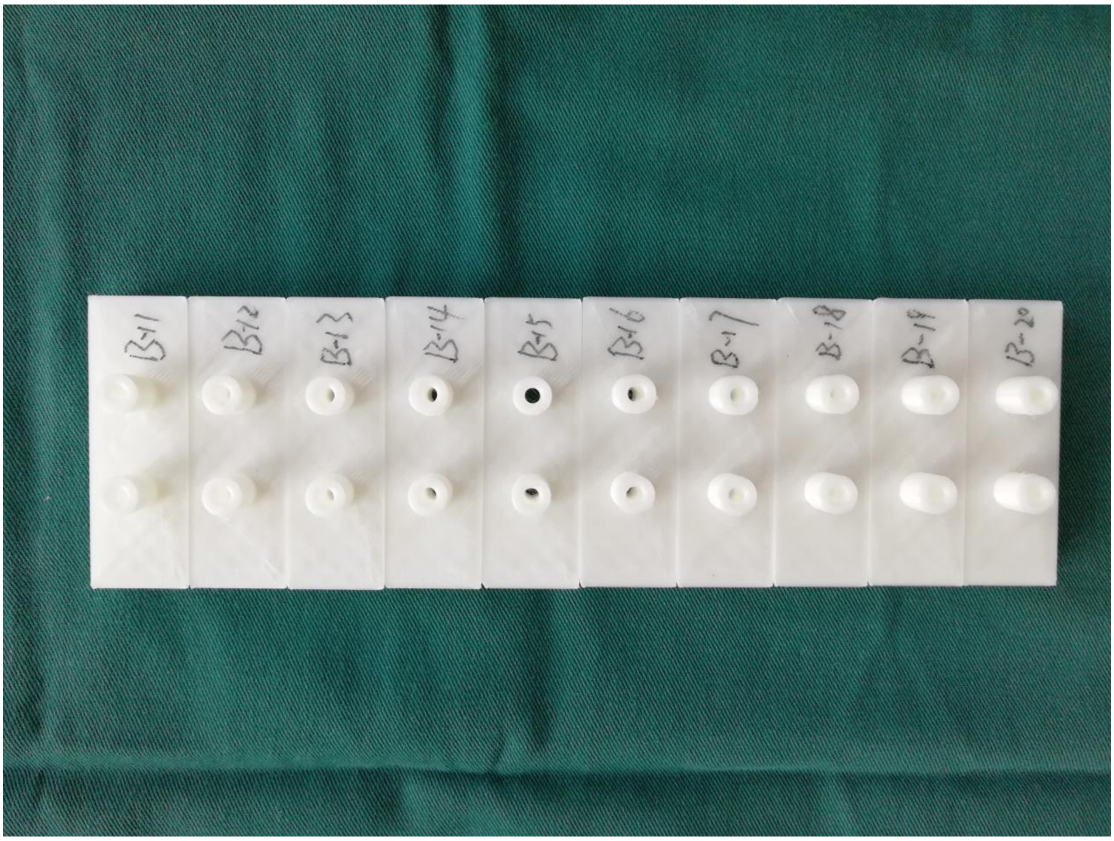

Figure S14. All modules of Group D.

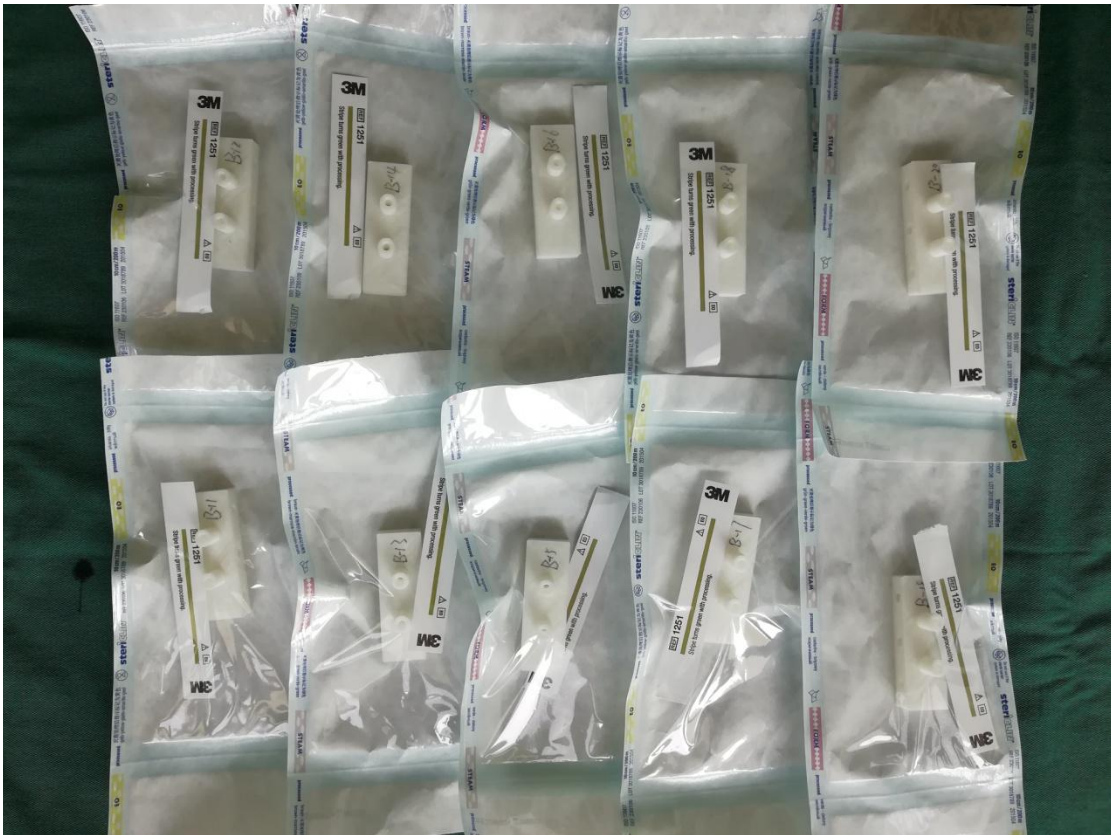

Figure S15. EO sterilization

Group E:

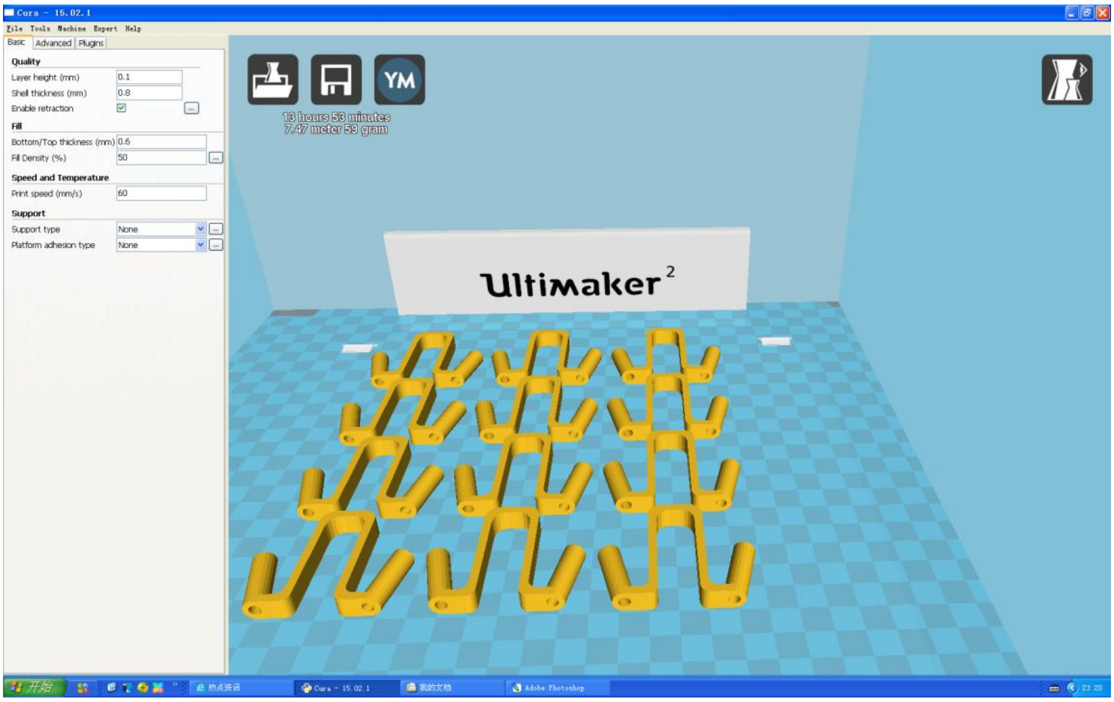

Figure S16. Slice using CURA software.

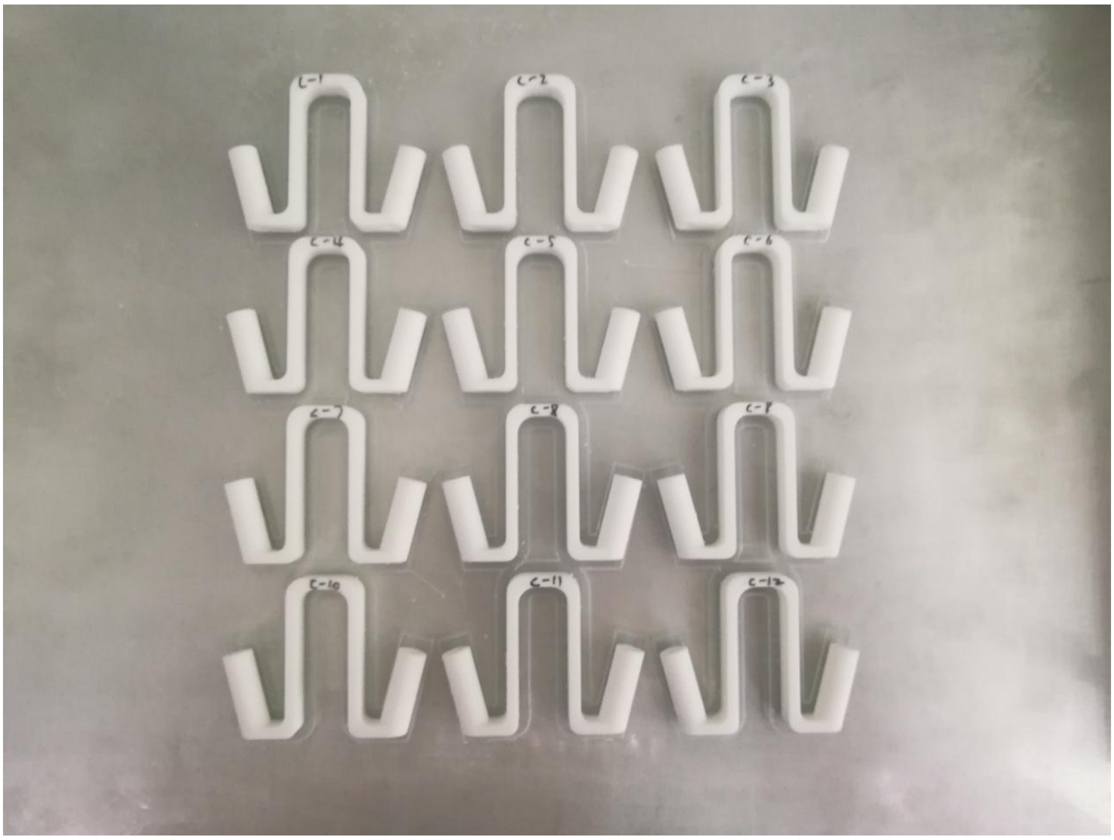
Figure S17. 3D printing was complete.

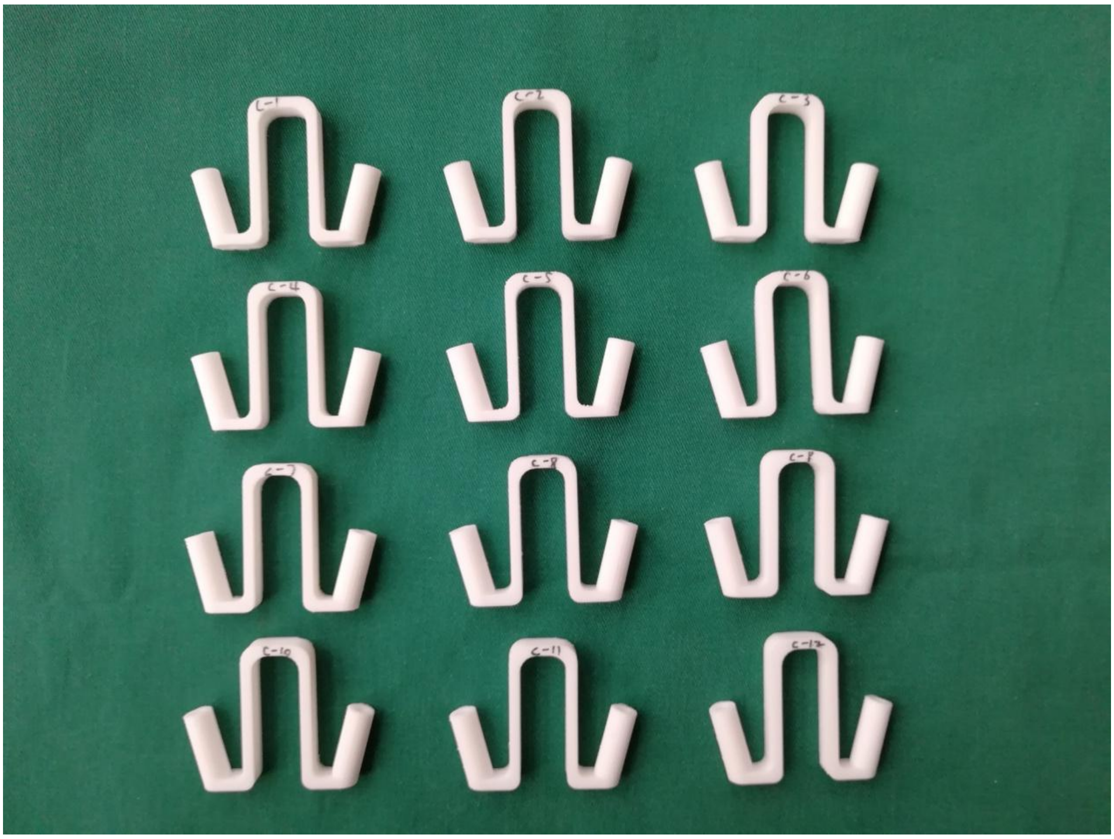
Figure S18. All modules of Group E.

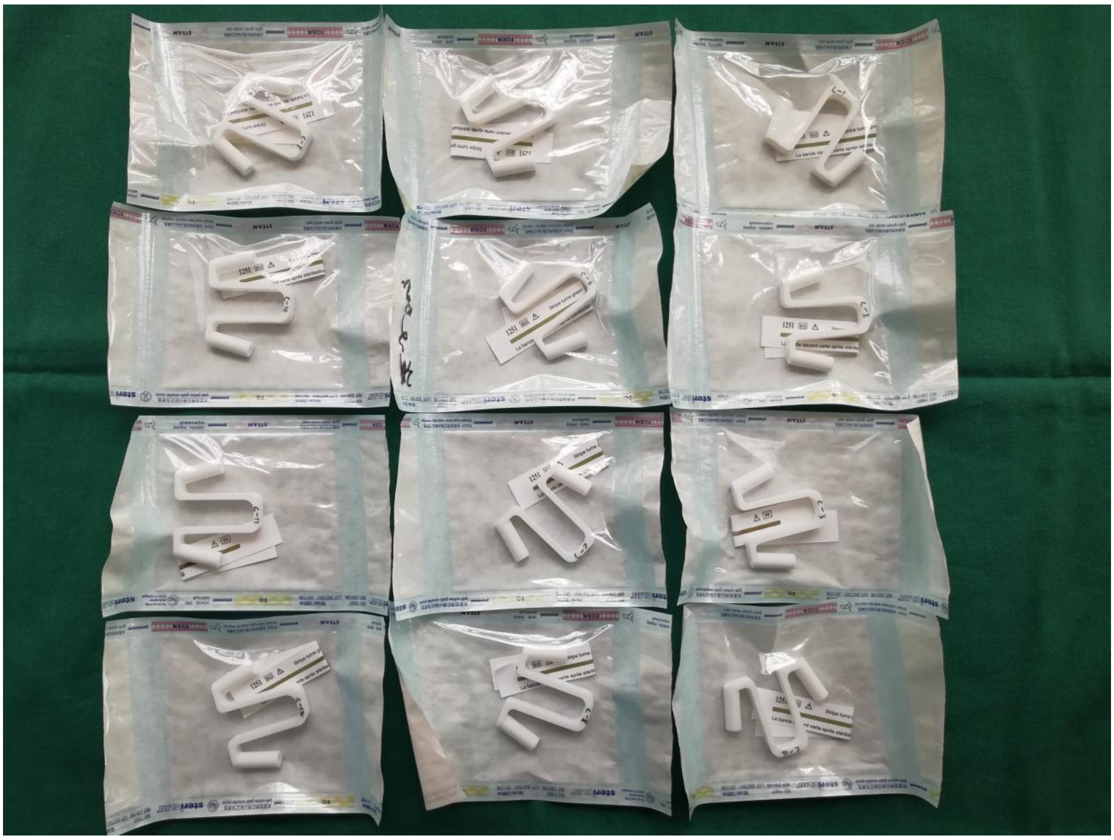

Figure S19. EO sterilization.

Experiment 3

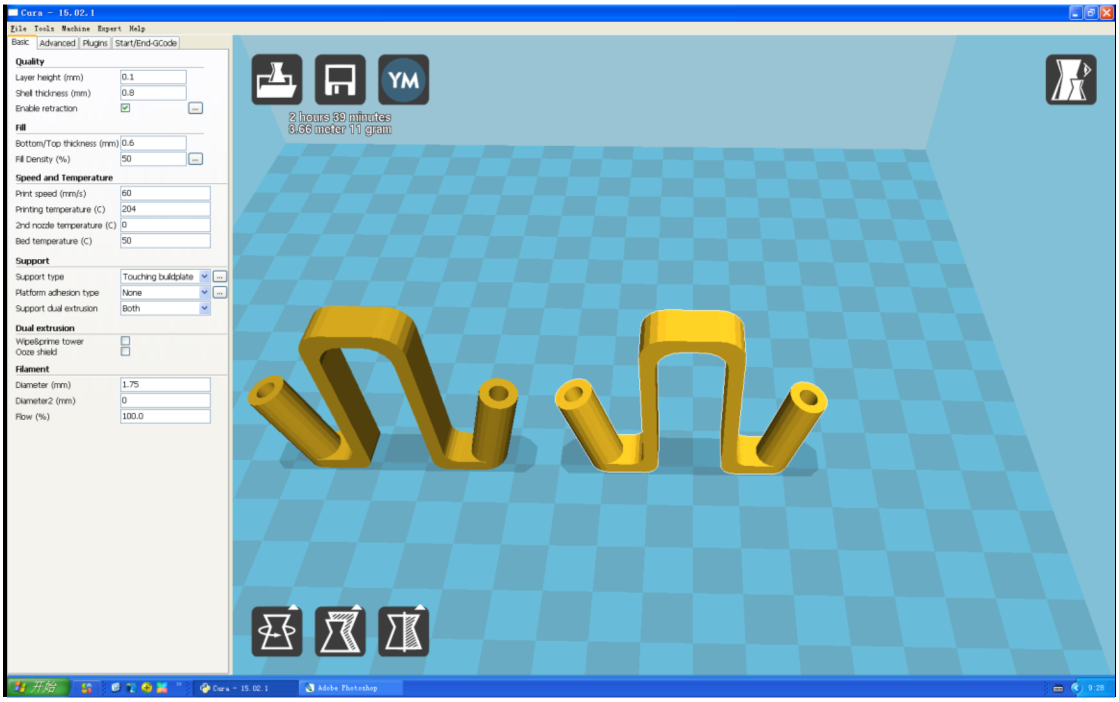

Figure S20. Slice using CURA software.

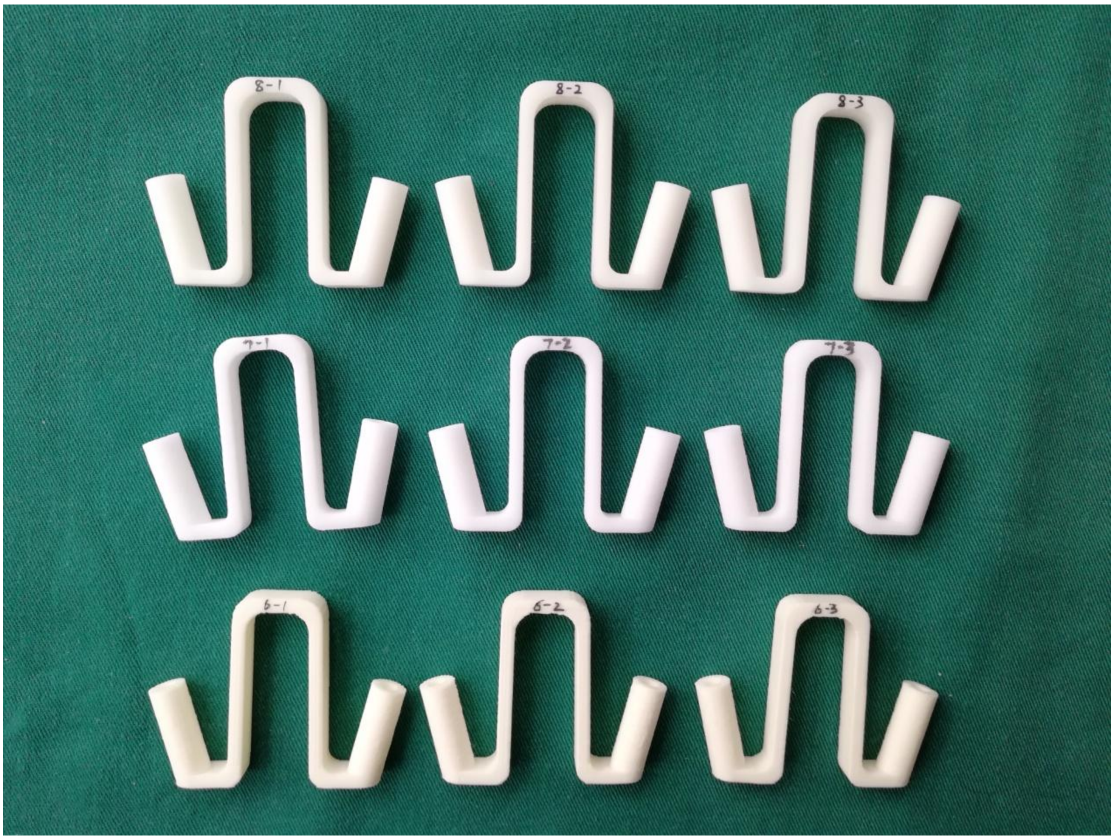

Figure S21. Before sterilization.

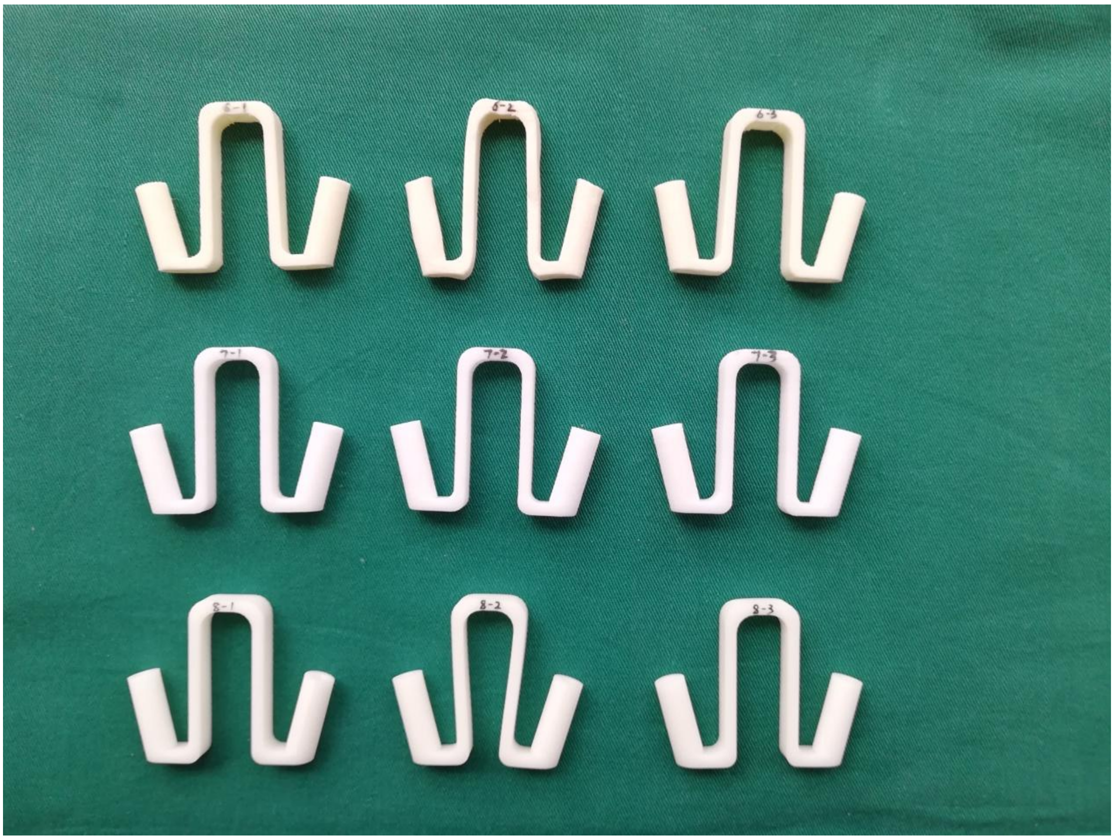

Figure S22. No deformation after EO sterilization(6-1,7-1,8-1) and plasma sterilization(6-3,7-3,8-3); Severe deformation after autoclaving(6-2, 8-2).

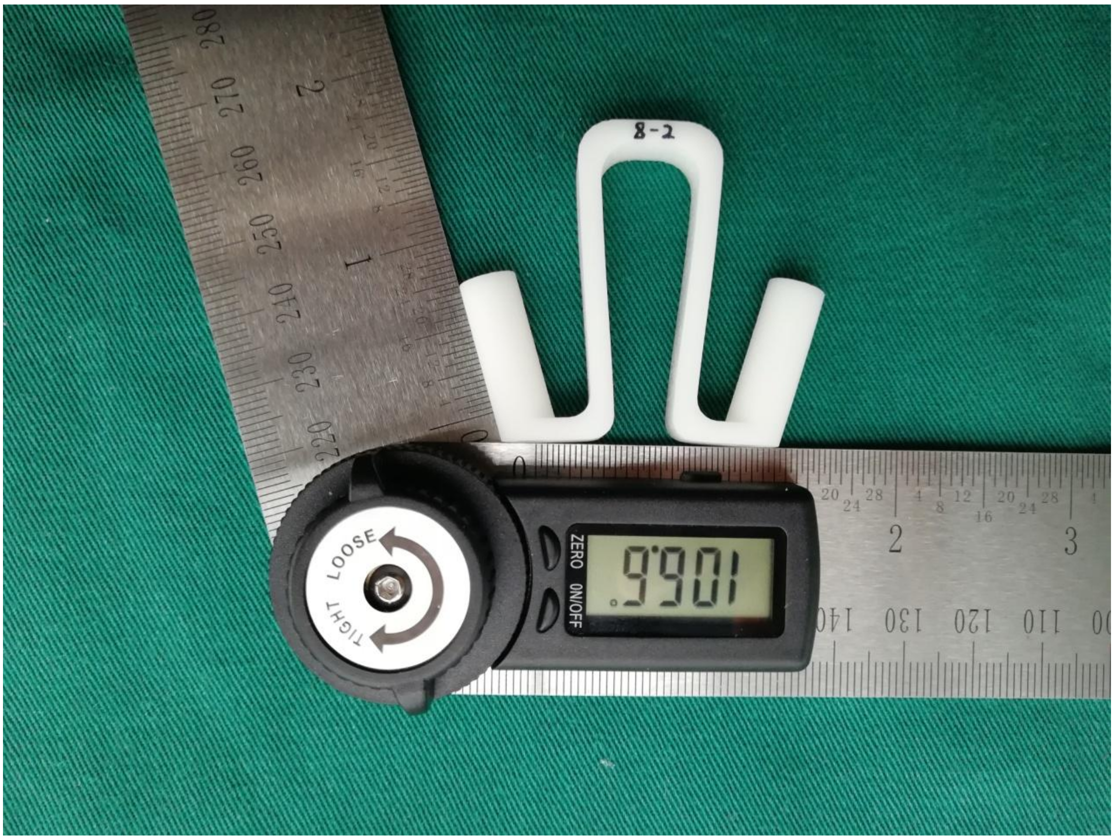

Figure S23. Obvious change in angle of guide hole.

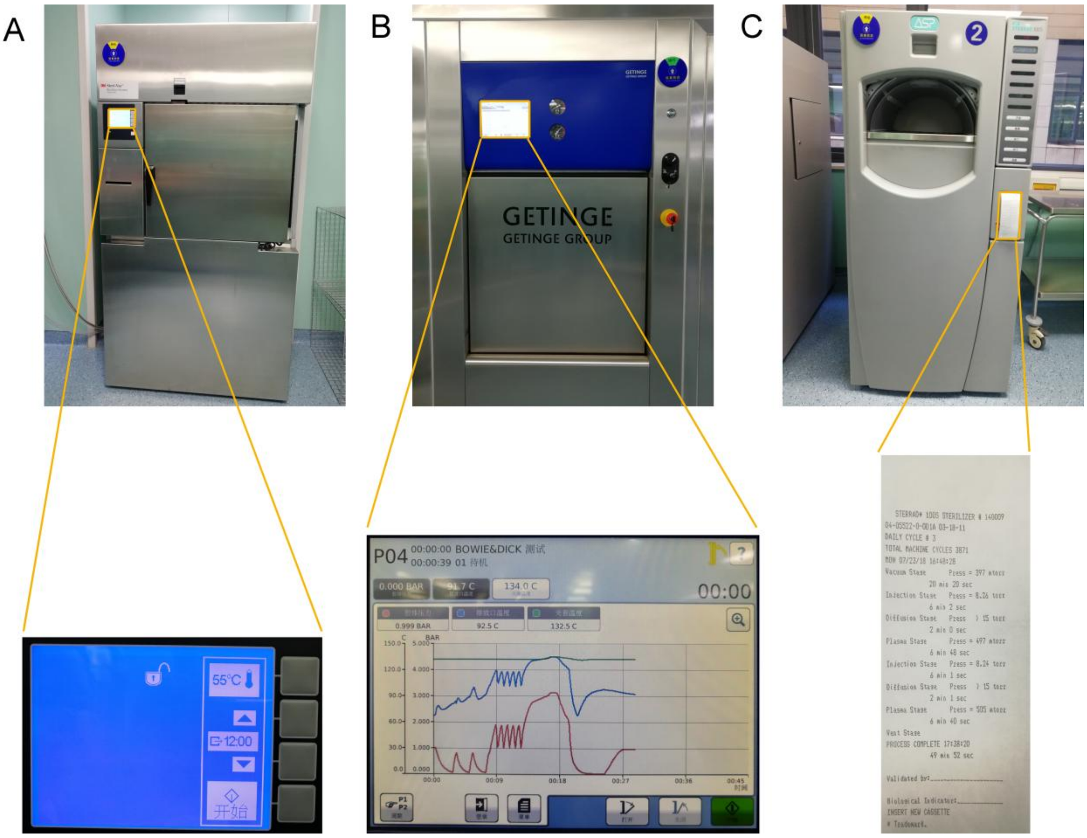

Figure S24. Methods and parameters of sterilization: (A) EO sterilization equipment; (B)

high-pressure steam sterilization equipment; (C) plasma sterilization equipment.

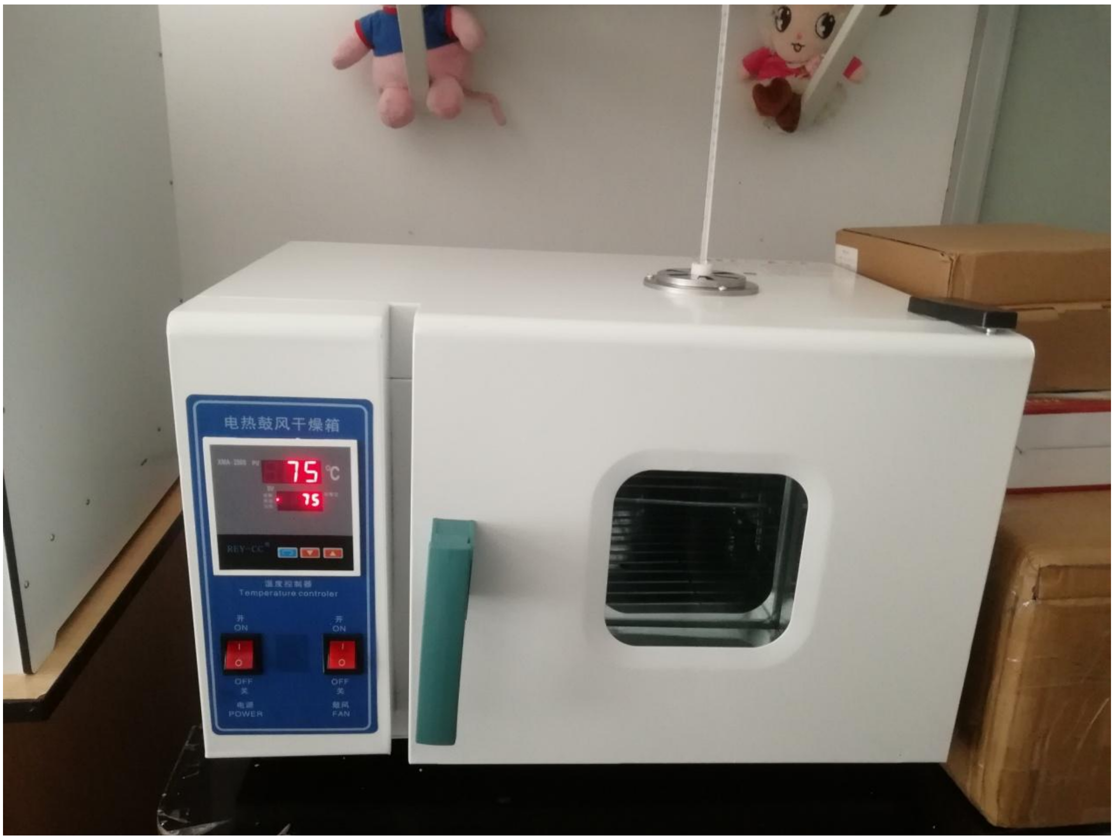

Figure S25. A constant temperature drying oven.

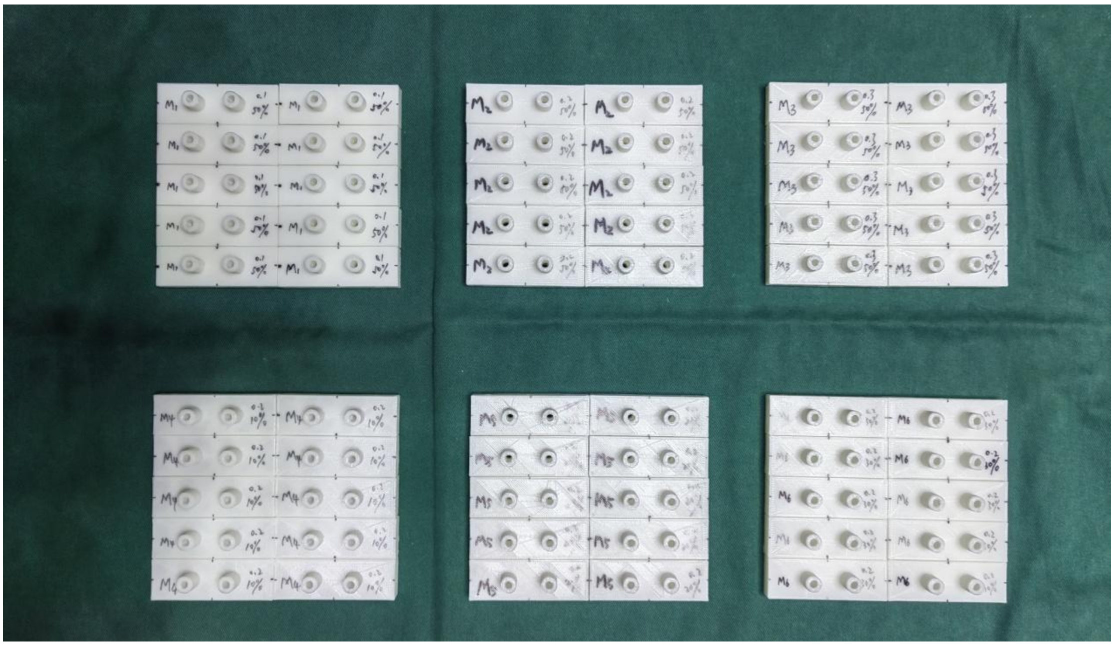

Figure S26. M1~M6 of M series.

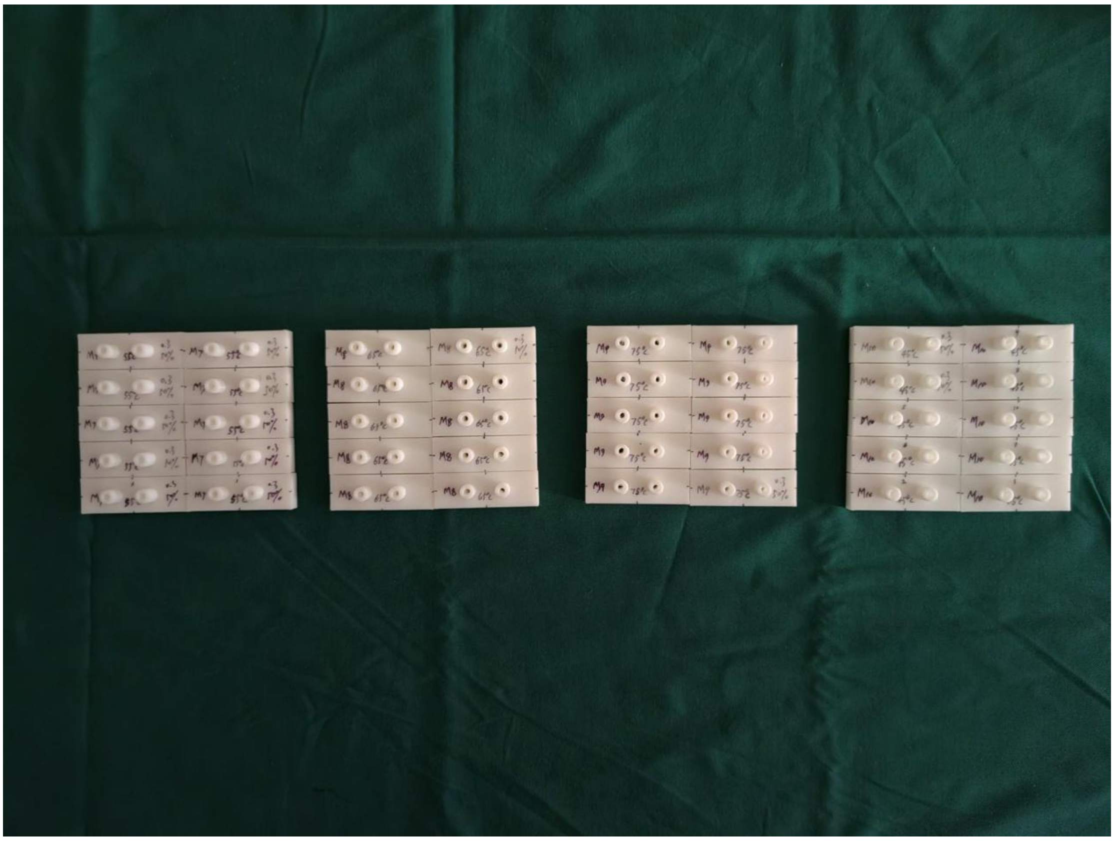

Figure S27. M7~M10 of M series.

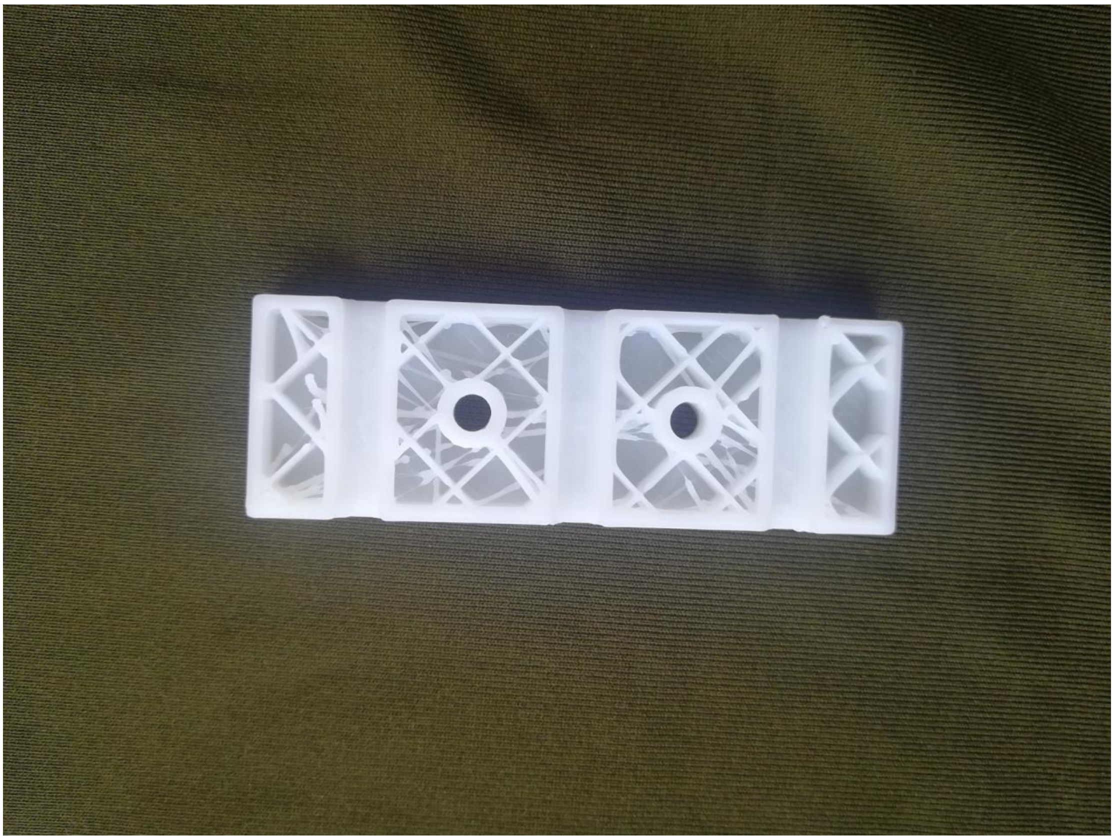

Figure S28. Infill pattern of the module

Original data 1

		Before sterilization			After sterilization		
Module size before and after EO sterilization （mm）		L1	L2	L3	L1	L2	L3
	A-1	59.92	20.00	9.98	58.90	19.60	10.20
	A-2	60.00	20.00	10.00	59.14	19.72	10.16
	A-3	59.90	19.96	10.00	59.00	19.58	10.16
	A-4	60.00	20.00	10.00	59.12	19.56	10.16
	A-5	60.00	19.98	9.98	58.80	19.62	10.16
	A-6	60.02	19.98	10.00	59.22	19.68	10.20
	A-7	59.94	19.90	10.02	59.00	19.50	10.30
	A-8	60.00	19.92	10.00	58.84	19.58	10.18
	A-9	59.96	19.94	10.00	58.78	19.60	10.24
	A-10	60.00	19.94	10.00	59.10	19.46	10.20
	B-1	60.22	20.12	9.92	59.10	19.74	10.06
	B-2	60.10	20.10	9.92	58.94	19.76	10.06
	B-3	60.20	20.20	9.96	59.22	19.70	10.18
	B-4	60.10	20.14	10.00	58.94	19.78	10.20
	B-5	60.18	20.14	10.00	59.02	19.76	10.18
	B-6	60.00	20.10	10.00	59.10	19.70	10.28
	B-7	60.10	20.16	9.96	59.00	19.80	10.12
	B-8	60.10	20.16	10.00	58.96	19.78	10.22
	B-9	60.10	20.16	9.92	59.00	19.82	10.08
	B-10	60.10	20.10	9.98	59.06	19.70	10.18
	A-11	60.00	20.22	10.00	59.14	20.44	9.88
	A-12	60.00	20.18	10.00	59.14	20.44	9.92
	A-13	60.02	20.20	10.00	59.14	20.42	9.92
	A-14	59.96	20.18	10.00	59.00	20.40	9.96
	A-15	60.00	20.20	10.04	59.00	20.40	10.00
	A-16	60.00	20.16	10.00	59.08	20.48	9.98
	A-17	59.90	20.18	10.02	58.98	20.40	9.96
	A-18	60.00	20.20	9.98	59.04	20.46	9.88
	A-19	60.00	20.20	10.00	58.98	20.40	9.88
	A-20	60.00	20.22	10.10	59.00	20.48	10.00
	B-11	59.88	19.98	10.00	59.80	20.00	10.00
	B-12	59.80	20.00	10.00	59.78	20.00	9.98
	B-13	59.84	20.00	10.02	59.68	20.00	10.00
	B-14	59.78	20.02	10.00	59.60	20.02	10.00
	B-15	59.90	20.00	10.02	59.76	20.00	10.00
	B-16	59.84	20.00	9.98	59.68	20.00	9.98
	B-17	59.80	20.00	10.00	59.64	20.02	10.00
	B-18	59.76	20.02	10.00	59.60	20.02	10.02
	B-19	59.84	20.00	10.00	59.68	19.98	10.00
	B-20	59.82	20.00	10.04	59.66	20.00	10.00
PLA+	A-21	60.14	20.22	10.04	58.44	19.62	10.30

	A-22	60.10	20.18	10.00	58.34	19.64	10.28
	A-23	60.10	20.22	9.96	58.38	19.66	10.26
	A-24	60.02	20.16	10.08	58.54	19.68	10.44
	A-25	60.06	20.22	10.10	58.30	19.68	10.48
	A-26	60.10	20.16	10.00	58.42	19.72	10.34
	A-27	60.14	20.18	10.06	58.48	19.68	10.30
	A-28	60.12	20.20	10.04	58.46	19.68	10.28
	A-29	60.00	20.22	9.98	58.44	19.72	10.30
	A-30	60.02	20.20	10.04	58.32	19.66	10.30

Original data 2 (M series)

	L1(pre-E0)					
	M1	M2	M3	M4	M5	M6
1	59.78	59.94	60.20	59.90	59.86	59.86
2	59.80	59.90	60.08	59.90	59.84	59.84
3	59.92	59.98	60.20	59.80	59.78	59.92
4	59.80	59.88	60.08	60.00	59.94	59.84
5	59.90	59.90	60.06	59.90	59.78	59.94
6	59.80	59.90	60.04	59.78	59.90	59.94
7	59.76	59.90	60.14	59.82	59.80	59.80
8	59.82	59.82	60.18	59.86	59.90	59.90
9	59.80	59.94	60.02	59.80	59.80	59.86
10	59.84	59.84	60.02	59.90	59.88	59.88

	L1(post-E0)					
	M1	M2	M3	M4	M5	M6
1	57.54	58.48	58.82	58.20	57.96	58.26
2	57.80	58.12	58.74	58.36	58.08	58.16
3	57.52	58.26	58.62	58.08	58.06	58.20
4	57.96	58.18	58.76	58.08	58.02	58.04
5	57.78	58.02	58.62	58.12	58.26	58.24
6	57.70	58.24	58.80	58.24	58.06	58.30
7	57.80	58.30	58.86	58.20	58.00	58.24
8	57.90	58.34	58.54	58.28	58.10	58.06
9	57.74	58.22	58.66	58.00	58.24	58.00
10	57.70	58.54	58.96	58.18	58.16	58.12

	L1(pre-)			
	M7	M8	M9	M10
1	60.12	60.12	60.04	60.20
2	60.18	60.24	60.16	60.06
3	60.22	60.00	60.04	60.14
4	60.08	60.08	60.00	60.20
5	60.10	60.06	60.14	60.00
6	60.00	60.20	60.10	60.04
7	60.02	60.06	59.98	60.04
8	60.06	60.02	60.08	60.06
9	60.14	60.04	60.16	60.20
10	60.24	60.18	60.10	59.98

	2h			
	M7	M8	M9	M10
1	59.56	59.14	58.56	60.20
2	59.66	59.14	58.42	60.10
3	59.32	59.08	58.62	60.16
4	59.38	59.08	58.60	60.20
5	59.64	59.06	58.64	60.08
6	59.44	58.98	58.52	60.06
7	59.68	59.10	58.50	60.06
8	59.50	58.90	58.58	60.04
9	59.60	59.00	58.44	60.18
10	59.68	59.04	58.66	59.98

	4h			
	M7	M8	M9	M10
1	59.46	58.84	58.58	60.20
2	59.42	59.04	58.54	60.10
3	59.52	58.92	58.52	60.14
4	59.48	58.92	58.54	60.16
5	59.60	58.90	58.44	60.02
6	59.32	59.02	58.44	60.08
7	59.52	58.98	58.54	60.04
8	59.54	59.02	58.46	60.00
9	59.36	59.00	58.58	60.18
10	59.60	59.04	58.62	60.00

	6h			
	M7	M8	M9	M10
1	59.44	59.06	58.56	60.20
2	59.32	59.02	58.50	60.06
3	59.42	58.90	58.44	60.18
4	59.48	58.98	58.62	60.20
5	59.32	58.98	58.44	60.06
6	59.56	59.00	58.62	60.04
7	59.50	59.06	58.54	60.06
8	59.60	58.86	58.54	60.04
9	59.52	59.08	58.64	60.16
10	59.46	59.04	58.64	59.98

	L2(pre-E0)					
	M1	M2	M3	M4	M5	M6
1	20.00	19.94	20.26	19.90	20.08	20.06
2	19.96	19.90	20.14	19.98	20.00	20.00
3	19.82	20.04	20.30	19.96	19.96	19.90
4	19.94	19.92	20.12	19.94	19.88	19.98
5	19.96	20.04	20.22	20.08	20.00	20.00
6	19.88	20.04	20.22	20.00	19.96	19.94
7	19.98	20.00	20.20	19.96	19.84	19.98
8	19.90	19.98	20.12	20.02	20.04	19.90
9	20.00	19.90	20.26	20.00	19.84	19.88
10	19.92	20.02	20.10	19.92	19.92	20.06

	L2(post-E0)					
	M1	M2	M3	M4	M5	M6
1	19.36	19.72	19.98	19.58	19.70	19.54
2	19.48	19.74	20.04	19.40	19.76	19.54
3	19.48	19.74	19.98	19.66	19.76	19.58
4	19.34	19.64	19.88	19.58	19.64	19.70
5	19.52	19.86	19.90	19.68	19.56	19.52
6	19.38	19.60	20.02	19.58	19.48	19.66
7	19.48	19.72	19.92	19.58	19.72	19.70
8	19.30	19.62	19.88	19.62	19.72	19.62
9	19.50	19.64	19.82	19.68	19.48	19.66
10	19.48	19.64	19.92	19.54	19.48	19.66

	L2(pre-)			
	M7	M8	M9	M10
1	20.16	20.26	20.16	20.22
2	20.26	20.04	20.24	20.16
3	20.22	20.20	20.14	20.30
4	20.20	20.28	20.26	20.06
5	20.28	20.18	20.22	20.14
6	20.26	20.24	20.20	20.22
7	20.16	20.18	20.22	20.20
8	20.24	20.12	20.28	20.22
9	20.28	20.20	20.08	20.24
10	20.08	20.26	20.22	20.24

	2h			
	M7	M8	M9	M10
1	20.06	19.96	19.72	20.20
2	19.94	19.98	19.74	20.16
3	20.06	19.76	19.78	20.28
4	20.06	20.00	19.82	20.08
5	20.06	20.04	19.80	20.18
6	19.98	19.92	19.82	20.24
7	20.10	19.98	19.84	20.22
8	20.00	19.86	19.72	20.24
9	20.06	19.94	19.80	20.30
10	20.14	19.92	19.68	20.26

	4h			
	M7	M8	M9	M10
1	19.92	19.80	19.84	20.24
2	19.94	19.94	19.68	20.14
3	20.10	19.90	19.78	20.28
4	20.04	19.90	19.74	20.08
5	20.06	19.88	19.80	20.14
6	20.06	20.00	19.80	20.26
7	20.14	20.00	19.82	20.24
8	20.06	19.90	19.82	20.24
9	20.02	19.92	19.74	20.28
10	19.92	19.92	19.68	20.28

	6h			
	M7	M8	M9	M10
1	19.98	19.80	19.74	20.22
2	20.02	19.94	19.84	20.16
3	19.98	19.92	19.76	20.28
4	20.04	19.94	19.84	20.08
5	20.04	20.00	19.80	20.16
6	20.02	19.88	19.80	20.24
7	20.04	19.98	19.72	20.24
8	19.92	19.88	19.82	20.24
9	20.10	20.00	19.68	20.30
10	20.10	19.94	19.78	20.26

	L3(pre-E0)					
	M1	M2	M3	M4	M5	M6
1	9.76	9.90	9.62	9.76	9.80	9.86
2	9.66	9.94	9.60	9.80	9.84	9.90
3	9.74	9.82	9.56	9.76	9.80	9.96
4	9.70	9.84	9.56	9.80	9.90	9.90
5	9.80	9.84	9.66	9.80	9.80	9.92

	L3(post-E0)					
	M1	M2	M3	M4	M5	M6
1	10.08	10.08	9.76	10.04	10.06	10.06
2	10.12	10.04	9.88	10.06	10.06	10.16
3	10.10	10.10	9.80	10.04	10.04	10.16
4	9.96	10.20	9.80	10.02	10.10	10.06
5	9.98	10.00	9.88	10.04	10.12	10.16

	L3(pre-)			
	M7	M8	M9	M10
1	9.60	9.66	9.58	9.64
2	9.60	9.72	9.66	9.62
3	9.64	9.64	9.66	9.66
4	9.60	9.60	9.72	9.68
5	9.60	9.60	9.66	9.68

	2h			
	M7	M8	M9	M10
1	9.70	9.92	9.94	9.66
2	9.76	9.84	10.06	9.60
3	9.74	9.88	9.94	9.62
4	9.68	9.90	9.96	9.70
5	9.70	9.78	9.90	9.74

	4h			
	M7	M8	M9	M10
1	9.68	9.84	9.86	9.66
2	9.66	9.84	10.02	9.60
3	9.66	9.84	9.96	9.64
4	9.66	9.84	9.92	9.66
5	9.68	9.84	9.94	9.74

	6h			
	M7	M8	M9	M10
1	9.68	9.94	9.94	9.66
2	9.72	9.94	10.00	9.60
3	9.68	9.86	10.06	9.64
4	9.70	9.90	10.02	9.74
5	9.70	9.82	10.06	9.70

6	9.66	9.90	9.56	9.76	9.90	9.94	10.08	10.10	9.80	10.02	10.04	10.10	9.58	9.72	9.58	9.62	9.66	9.86	10.00	9.60	9.68	9.86	10.04	9.62	9.66	9.84	10.00	9.64
7	9.74	9.84	9.64	9.78	9.78	9.96	10.08	10.14	9.78	10.06	10.12	10.06	9.58	9.64	9.62	9.62	9.64	9.82	9.98	9.64	9.68	9.78	10.00	9.64	9.70	9.90	10.00	9.64
8	9.72	9.86	9.64	9.80	9.80	9.86	10.10	10.08	9.82	10.06	10.12	10.12	9.66	9.62	9.64	9.68	9.64	9.84	10.02	9.70	9.70	9.86	9.98	9.68	9.74	9.84	9.92	9.70
9	9.64	9.90	9.52	9.74	9.82	9.94	10.04	10.16	9.78	10.00	10.06	10.20	9.54	9.68	9.72	9.68	9.68	9.86	9.90	9.70	9.72	9.92	9.90	9.68	9.66	9.86	9.98	9.68
10	9.76	9.80	9.62	9.74	9.84	9.86	9.98	10.12	9.82	10.04	10.14	10.08	9.70	9.70	9.64	9.66	9.66	9.80	9.98	9.68	9.76	9.82	10.00	9.68	9.62	9.82	9.90	9.68
